# Transcending Lipophilicity: Design of Oxotriphenylhexanoate (OTHO) Hydrogels Decorated With 9,10‐Diphenylanthracene Fluorescent Probes via Late‐Stage Polarity Swap

**DOI:** 10.1002/open.70309

**Published:** 2026-09-23

**Authors:** Michel Taheri, Mario Martos, Olivia Lundgren, Henrik Sundén

**Affiliations:** ^1^ Department of Chemistry and Molecular Biology University of Gothenburg Gothenburg Sweden

**Keywords:** 9,10‐diphenylanthracene, hydrogels, low molecular weight gels, oxotriphenylhexanoates, polarity swap

## Abstract

Low molecular weight gels are versatile materials prized for their ease of modification compared to their polymeric counterparts, which facilitate their valorization into task‐specific materials. However, such modifications seldom extend beyond mere functionalization, and strategies for transferring privileged gelator scaffolds between hydrophilic and lipophilic environments remain rare. In this work, we present a general approach for the late‐stage polarity swap (LSPS) of the lipophilic, highly modular oxotriphenylhexanoate (OTHO) scaffold after functionalization with the fluorescent probe 9,10‐diphenylanthracene (DPA). The compounds obtained were tested for gelation under different conditions, with the resulting hydrogels characterized by oscillatory rheology. SEM images of the xerogels reveal fibrillar assemblies, demonstrating the self‐assembling capabilities of OTHOs remain unaffected regardless of medium.

## Graphical abstract

Transferring robust organogelator cores to water is a promising approach to extend the scope and simplify the preparation of functional hydrogels, of interest in biomedical applications. We demonstrated this strategy by obtaining 9,10‐diphenylanthracene‐decorated oxotriphenylhexanoate hydrogels through organic solvent–based chemistry followed by late‐stage polarity swap. The gels were then subjected to mechanical and morphological analysis.

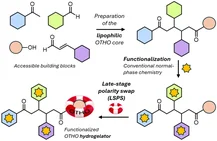

## 1 Introduction

**FIGURE 1:**
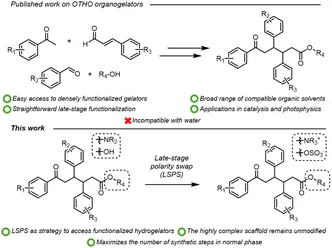
Schematic representation of the LSPS approach applied to OTHO gels.

Low molecular weight gelators (LMWGs) are a breed of molecules capable of self‐assembly, which have gained notable attention over the last two decades. Gels from LMWGs are categorized as physical gels, in which supramolecular interactions drive the assembly of the gelator into a network [1]. Physical gels have been known for more than two centuries; however, until relatively recently, research had mostly focused on polymeric gels, comprised of long chains associated through noncovalent interactions [2]. In contrast, gels from LMWGs are formed from relatively small molecules (200–1000 Da) that assemble into macroscopic structures via hierarchical self‐assembly [3, 4]. This feature constitutes a key advantage, as their molecular size is compatible with conventional small‐molecule synthesis, making modification and functionalization a much more straightforward and precise affair. Consequently, LMWGs represent an attractive platform for the development of task‐specific materials [5]. Indeed, many different LMWGs modified with endless functionalities have been developed over the years, with examples targeting catalysis [6], energy storage [7], or biomedical applications including drug delivery [8]. A comparatively underexplored modification, however, is the alteration of polarity of a gelator core to enable gelation in an otherwise incompatible solvent [9, 10]. This can be mostly attributed to the often detrimental effect modifications have on gelation properties. Nevertheless, there are benefits to this approach, especially when it comes to organo‐ to hydrogel transition. The synthesis and functionalization of lipophilic molecules are generally easier for the average lab owing to broader reagent and solvent compatibility as well as access to normal‐phase chromatographic purification. By contrast, many hydrogelators contain plenty of potentially reactive functionalities (e.g., hydroxyls, amines, and amides) [11]. In this context, the possibility of integrating water compatibility through late‐stage polarity swap (LSPS) in the preparation of functional hydrogelators not only simplifies the process but also broadens the spectrum of accessible gelator scaffolds (Figure 1).

This concept has recently been applied to, for example, pyridine‐decorated hydrazone and semicarbazone LMWGs [9]. By generating the corresponding pyridine N‐oxides, water gelation could be achieved. A few years earlier, our group reported a related strategy for the oxotriphenylhexanoate (OTHO) scaffold, in which quaternary ammonium salts and pyridine N‐oxides were employed to turn this highly lipophilic molecule into an effective hydrogelator [12]. However, in both protocols, the primary focus was on achieving water solubility, with no further modification enabling new functionality in the resulting hydrogels. In this work, we present a different strategy for the polarity swap of OTHO derivatives incorporating a 9,10‐diphenylanthracene (DPA). OTHOs, first introduced a little over a decade ago, represent a prominent class of LMWGs boasting excellent synthetic flexibility and high compatibility with common small‐molecule chemistry [13]. This has led to their implementation in diverse fields, including catalysis [6], photophysics [14, 15, 16], and singlet‐oxygen release [17]. Because DPA is a lipophilic polyaromatic structure with numerous applications, including triplet–triplet annihilation [14] and endoperoxide formation [17], it represents an ideal yet challenging starting point for evaluating the robustness and functional adaptability of this hydrogel system.

## 2 Results and Discussion

### 2.1 Preparation of Hydrophilic PA‐OTHO Gels

**SCHEME 1:**
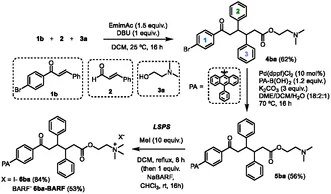
Preparation of the cationic PA‐OTHOs **6ba** and **6ba‐BARF**.

Based on our previous experience on the preparation of OTHO hydrogels, we decided to explore a tertiary ammonium salt as hydrophilic component. The PA (9‐phenylanthracene) functionality was introduced at ring number 1 (Scheme 1) at the *para* position, in accordance with our findings during a compatibility study on the substitution of OTHOs [18]. Thus, we prepared the corresponding Br‐OTHO derivative via straightforward multicomponent OTHO reaction in good yield. Subsequent introduction of the PA unit through a Suzuki coupling, followed by methylation with methyl iodide, afforded the cationic PA‐OTHO **6ba** in good overall yield (Scheme 1).

**SCHEME 2:**
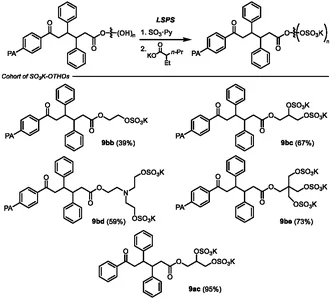
Sulfate‐decorated PA‐OTHOs **9bb–** **9be** and **9ac**. PA, phenylantracene. Reaction conditions: SO_3_·Py (2 equiv. per OH), MeCN, reflux, 1.5 h, then potassium 2‐ethylhexanoate (5 equiv. per SO_3_), EtOH, 4 °C, 30 min. Isolated yields after LSPS step.

However, **6ba** was completely insoluble in water, even after exchanging the iodide counter‐ion for the bulky **BARF**. A plausible explanation is that the PA functionality imparts excessive lipophilicity, preventing compound **6** from acting as a hydrogelator upon the introduction of only a single charged (cationic) group. This observation highlights the delicate equilibrium required for gelation, often described as riding a knife’s edge between solubility and insolubility [19]. Thus, we decided to change our strategy to promote water solubilization more reliably. Inspired by surfactant chemistry, we decided to use sulfates, which are both strongly hydrophilic and easily accessed from hydroxyl groups using relatively mild, selective, and well‐understood chemistry. We thus prepared a series of PA‐OTHOs from the corresponding Br derivatives using different alcohols, which were then subjected to sulfation, followed by ion exchange with potassium to afford compounds **9bb–** **9be** (Scheme 2).

As it turns out, the sulfation strategy worked well, and compounds **9bb**, **9bc**, and **9be** readily dissolved in water upon heating. Interestingly, despite bearing two highly hydrophilic sulfate groups, the amino‐derivative **9bd** was completely insoluble.

To determine the gelation capabilities of the PA‐OTHO sulfates, two sets of 0.5 wt./v.% solutions were prepared in deionized water. These samples were either subjected to sonication, a known gelation trigger for OTHOs [18], or left undisturbed. In cases where no gelation was observed, concentration was increased in increments of 0.5 wt./v.% until either precipitation or gelation occurred (Table 1).

**TABLE 1:** Gelation tests on sulfate PA‐OTHOs in deionized water.

[OTHO], wt./v.%	9bb	9bc	9bd	9be
0.5	S	S	I	S
1.0	I	S	—	S
1.5	—	P	—	S
2.0	—	—	—	S
3.0	—	—	—	S

No gelation was observed in any case. Compounds **9bb** and **9bc** formed precipitates, whereas **9be** remained in solution up to a concentration of 3.0 wt./v.%. However, a pronounced increase in viscosity was observed upon dissolution of **9bc**, even at low concentration.

Based on this, we theorized that primary assembly (i.e., individual molecule‐to‐molecule connection) might occur, but the resulting oligomeric assemblies were not able to associate in a gel‐like network. Instead, they aggregated and precipitated once their solubility limit was exceeded. It is well established that some hydrogels are very sensitive to the ionic composition of the medium, with alginate being a prime example [20]. We therefore hypothesized that ions might mediate a secondary assembly by bridging oligomeric supramolecular structures through electrostatic interactions to achieve gel network formation. Accordingly, we reexamined gelation behavior in the presence of different ions (Table 2). Indeed, marked differences from the initial tests were observed. K^+^ and Ca^2+^ triggered gelation for **9bc**, forming stable cloudy gels at room temperature after a few minutes (Figure S1A). Mg^2+^ generated large clumps of precipitate in the solution, which may be attributed to excessively strong or close coordination. Li^+^ and Na^+^ seemed to slightly favor solubility in all cases, although not enough to fully solubilize **9bb** and **9bd**. **9be** remained fully dissolved in all cases. Judging by the negligible differences observed among the potassium salts used, there does not seem to be any significant influence from the anion in the gelation process itself.

**TABLE 2:** Gelation tests on sulfate‐PA‐OTHOs (1.0 wt.v.%) in the presence of ions (0.05 M).

Salt	9bb	9bc	9bd	9be
LiCl	I	S	I	S
NaCl	I	S	I	S
KCl	I	G	I	S
MgCl_2_	I	P	I	S
CaCl_2_	I	G	I	S
KBr	I	G	I	S
KI	I	G	I	S
KOAc	I	G	I	S

Abbreviations: G, gel; I, insoluble; P, precipitate; S, clear solution.

Given the results obtained, we decided to focus our efforts on glycerol‐derived **9bc**. The unsubstituted **9ac** was prepared as a reference to assess the overall effect of the PA functionalization in the polarity‐swapped OTHO by testing gelation in the presence of K^+^ and Ca^2+^ (Table 3).

**TABLE 3:** Gelation tests on **9ac** and **9bc** (1.0 wt.v.%) in the presence of ions (0.05 M).

Salt	9ac	**9bc** a
None	P	S
KCl	P	G
CaCl_2_	G	G

a Results from Table 2.

There is a clear influence of the presence of the PA unit in gelation; while **9bc** formed gels in the presence of both ions, with Ca^2+^ leading to a visually weaker and more heterogeneous gel than its K^+^ counterpart, the unsubstituted **9ac** only gelated the Ca^2+^ solution, with K^+^ quickly leading to precipitation.

### 2.2 Rheology

**FIGURE 2:**
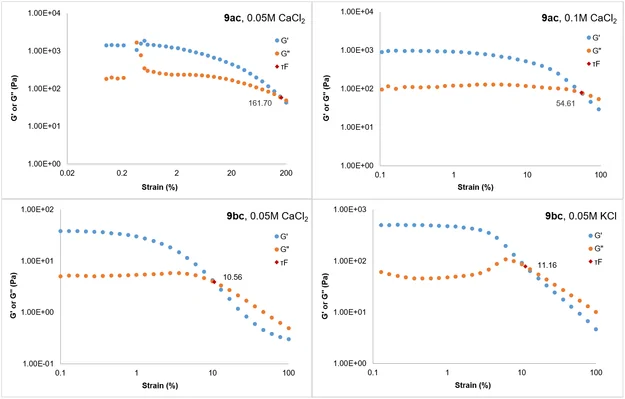
Amplitude sweeps (0.1–100/200% strain, 6.28 rad/s) of **9ac** and **9bc** gels in the presence of CaC_l2_ (0.05, 0.1 M) and KCl (0.05 M). Flow points (*τ*_F_) indicated in strain %.

Oscillatory rheology was used to study the mechanical properties of the gels. We first performed amplitude sweeps at constant frequency to evaluate the response of the materials and determine their linear viscoelastic (LVE) region (Figure 2). For **9ac**, an additional sample of gel with twice as much Ca^2+^ (0.1 M) was prepared to observe the effect of concentration. In both **9ac** samples, the response of the material follows essentially the same trend, with a long LVE and relatively early yield point (ca. 1% strain) but surprisingly high flow points of 162 and 55% strain, respectively. The values of G′ are relatively high and consistent with what is expected on a LMWG (one order of magnitude between G′ and G″) [16], although noticeably lower in the gel with the higher Ca^2+^, which could be traced to a larger fiber size. The results from **9bc** confirmed our empirical observations during the ion screening. Again, both gels present a very similar profile, but the KCl gel is much stronger, with G′/G″ values an order of magnitude higher and broader LVE than its CaCl_2_ counterpart, although with similar flow points. The latter gel collapsed after sampling, separating in blobs of precipitate and flowing water, thus being discarded from further mechanical testing.

**FIGURE 3:**
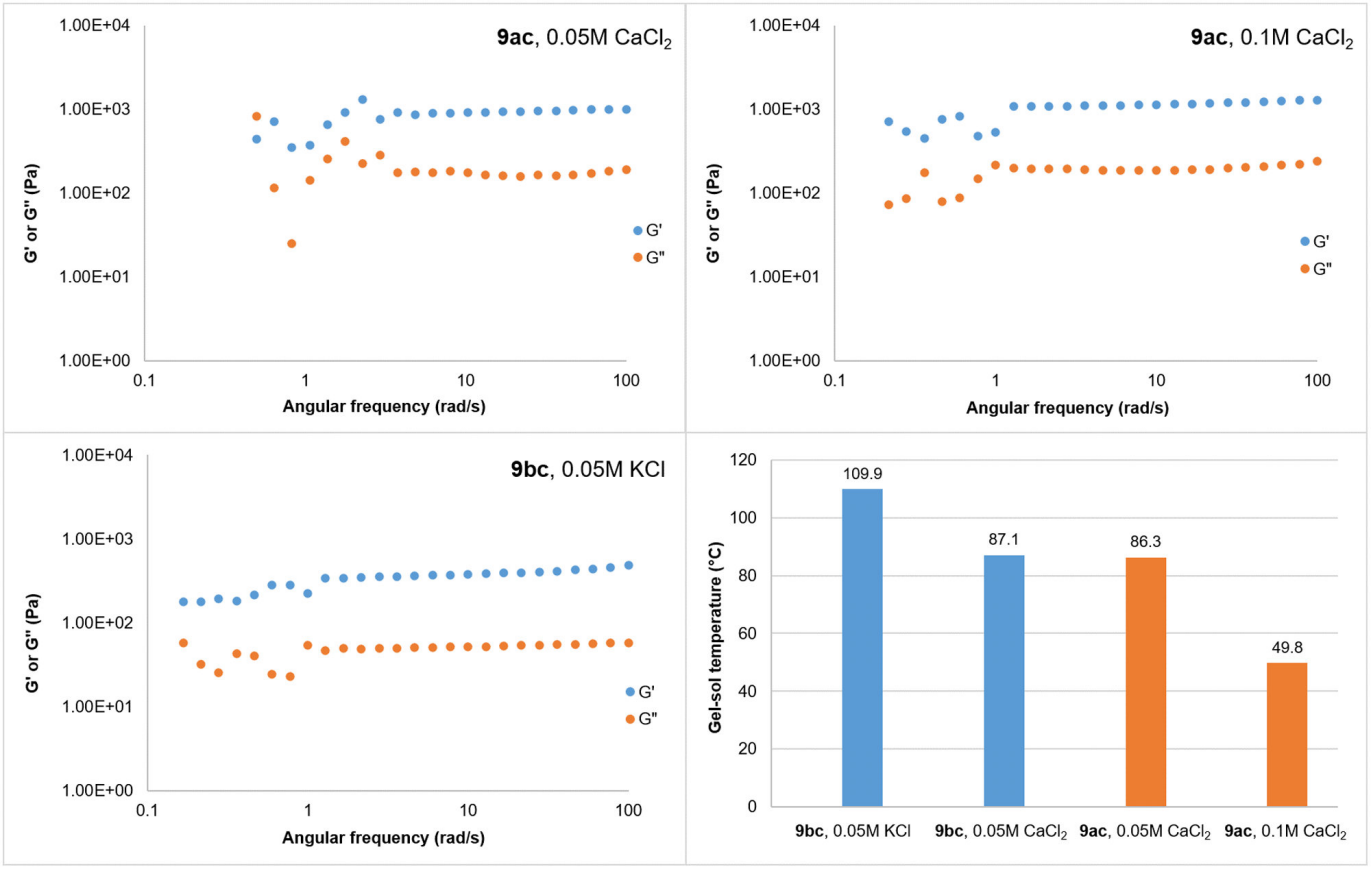
Frequency sweeps (0.1–100 rad/s, 0.5% strain) of **9ac** and **9bc** gels in the presence of CaCl_2_ (0.05, 0.1 M) and KCl (0.05 M) and *T*_gel‐sol_ of the **9ac** and **9bc** gels.

Next, we investigated the frequency dependence of the materials under constant strain (Figure 3). Both **9ac** gels were essentially frequency independent over the investigated frequency range. Although equilibration times become increasingly longer under 1 rad/s, there is no trend towards liquefaction at low frequency. The KCl **9bc** gel presents a more stable but equally independent behavior. Compared to OTHO gelators in general, the LSPS approach therefore does not appear to substantially influence the rheological response [6, 12, 17, 18], which remains broadly comparable to that of OTHO organogels, especially with respect to frequency dependence. **9ac** gels are particularly mechanically stable, exhibiting properties exceeding those of our strongest boronic acid–reinforced OTHOs [16].

We also determined the gel‐sol transition temperature (*T*_gel‐sol_), and the observed thermal stability trends are broadly consistent with the amplitude sweeps results. The KCl **9bc** gel shows the highest transition temperature, with a 23 °C difference relative to its 0.05 M CaCl_2_ counterpart (Figure 3). For **9ac**, the 0.05 M gel exhibited an almost identical *T*_gel‐sol_ to that of the corresponding **9bc** gel. Interestingly, there is a very pronounced decrease in *T*_gel‐sol_ for the 0.1M CaCl_2_ gel, which shows the least thermal stability of all materials.

### 2.3 Scanning Electron Microscopy

At this point, dried gel samples were examined by scanning electron microscopy (SEM) to identify morphological features that could help rationalize the results of the rheological and thermal analyses.

**FIGURE 4:**
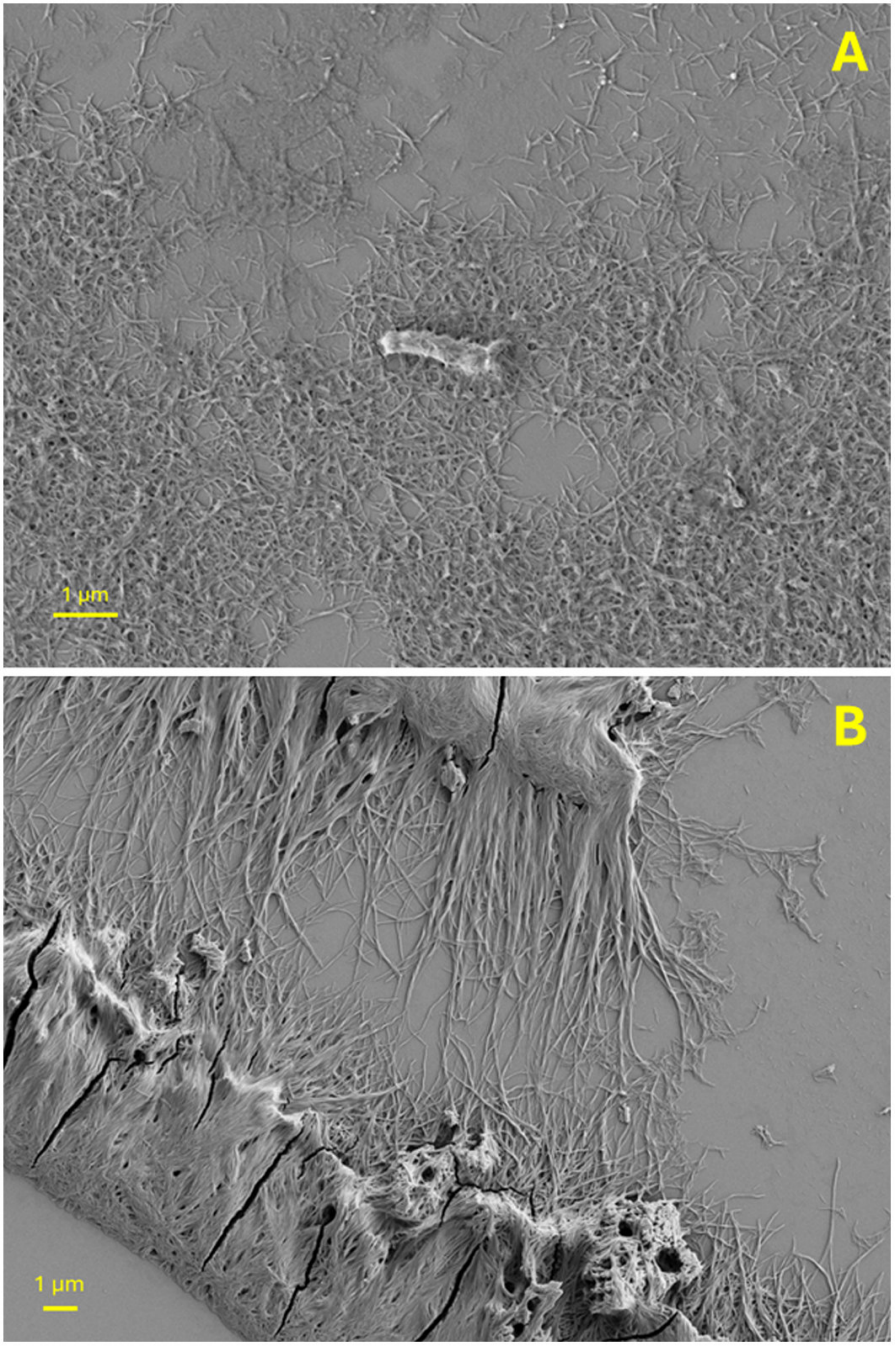
SEM images of **9ac** gels in (A) 0.05 M CaCl_2_ and (B) 0.1 M CaCl_2_.

The SEM images of both **9ac** gels show clearly defined fibrillar structures with similar morphology but important differences in thickness and length. The 0.05 M CaCl_2_ gel (Figure 4A) presents bundles of fibers roughly 1 µm long with a mean thickness of 33 nm, whereas its 0.1 M counterpart (Figure 4B) shows much longer (ca. 10 µm) and noticeably thicker (48 nm on average) fibers. This observation is consistent with the higher concentration of ions enabling the formation of larger structures through enhanced secondary assembly. Regarding rheology, the thinner fibers of the 0.05 M gel should provide a more intertwined and flexible network, which explains the slightly higher G′ and G” values observed. Conversely, the lower degree of interlocking between the fibers in the 0.1 M gel may contribute to its low thermal stability, potentially due to thermally induced disentanglement rather than disruption of the underlying intermolecular interactions.

**FIGURE 5:**
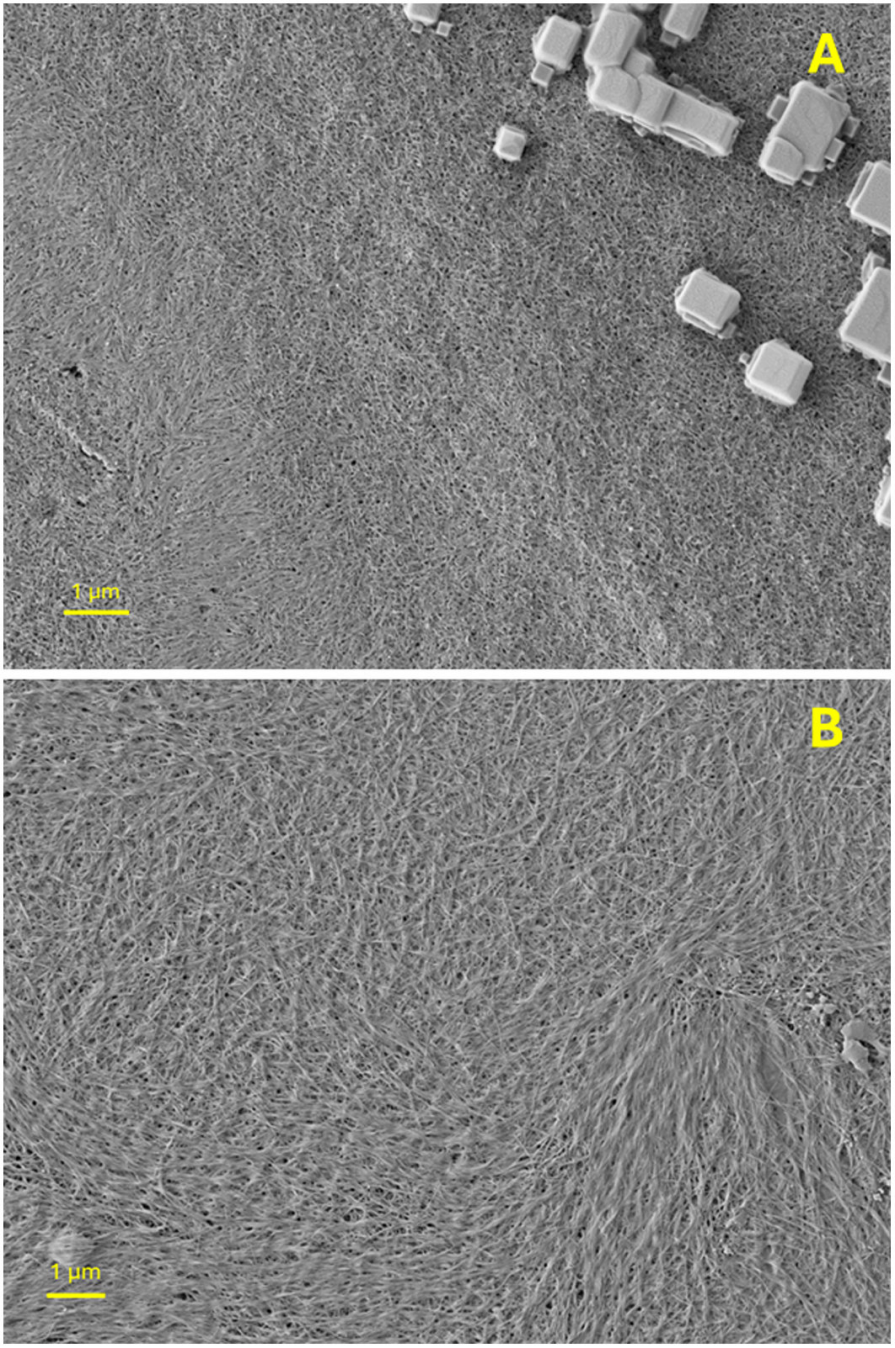
SEM images of **9bc** gels in (A) 0.05 M CaCl_2_ and (B) 0.05 M KCl.

The gels of **9bc** also present fibrillar structures, although their morphology is markedly different owing to the different cations used. The 0.05M CaCl_2_ gel (Figure 5A) presents relatively long fibers (several µm) with a mean thickness of 30 nm and heavily interlocked regions. The 0.05 M KCl gel presents a similarly interlocked structure with much thinner fibers (ca. 22 nm). The striking difference in strength between the two gels may arise from Ca^2+^ driving assembly too effectively, leading to the formation of fiber aggregates loosely held together within a thin gel matrix. This interpretation is consistent with the collapse and phase separation upon sampling as well as the very low values of G′ and G″ observed. This separation nevertheless appears to be mechanically triggered, given the high *T*_gel‐sol_ observed. FTIR analysis of both **9bc** xerogels does not reveal any significant deviation from that of the pure compound beyond the CaCl_2_ xerogel being strongly hygroscopic (Figure S20). This, along with the crystals observed in SEM, suggests that the interaction between the salts and OTHOs takes place exclusively during gelation, separating upon the removal of solvent into two discrete components.

## 3 Conclusion

We have demonstrated an LSPS approach for the straightforward preparation of functional hydrogels through the modification of the OTHO scaffold. We were able to successfully enable gelation in water after the insertion of the highly lipophilic PA functionality, obtaining fluorescent hydrogels. We also investigated the influence of ions in the gelation process, aiming towards triggerable systems where ions can be injected into OTHO solutions (and vice versa) to induce gelation at will. We measured the mechanical behavior of the materials through rheology, as well as their thermal stability, and correlated both with SEM imaging of the fibers comprising the gel structures. The results obtained here pave the way for a new breed of task‐specific OTHO hydrogels, merging the advantageous modular synthesis of these LMWGs with an aqueous matrix that broadens their applicability in a number of areas, for example, for use in biomedical applications.

## Funding

This study was supported by Svenska Forskningsrådet Formas (2025‐02663), vetenskapsrådet (2024‐04788), Marie Skłodowska‐Curie Actions postdoctoral fellowship (HORIZON‐MSCA‐PF OTHOx – g.n 101152118), Martina Lundgrens foundation (2025‐GU‐4994), and Vetenskapsrådet (2024‐04788).

## Conflicts of Interest

The authors declare no conflicts of interest.

## Supporting information

Supplementary Material

## Data Availability

The data that support the findings of this study are available in the supplementary material of this article.
