# Supplementary material for: Transcending Lipophilicity: Design of Oxotriphenylhexanoate (OTHO) Hydrogels Decorated With 9,10‐Diphenylanthracene Fluorescent Probes via Late‐Stage Polarity Swap
Source: ChemistryOpen. 2026 Sep 23;15(10):e70309. doi: 10.1002/open.70309 (PMC13601729; doi:10.1002/open.70309)
Supplement: Supplementary file 1 [file OPEN-15-e70309-s001.pdf]

Transcending Lipophilicity: Design of Oxotriphenylhexanoate (OTHO) Dydrogels Decorated with 9,10-Diphenylanthracene Fluorescent Probes via Late-Stage Polarity Swap.

*Michel Taheri‡, Mario Martos‡, Olivia Lundgren, Henrik Sundén**

‡Both authors contributed equally

Department of Chemistry and Molecular Biology, University of Gothenburg, Gothenburg, Sweden

E-mail: henrik.sunden@chem.gu.se

Supporting Information

Table of Contents

1. General information 2
2. Gelation and synthetic protocols 2
   Procedure for the preparation of **6ba** and **7ba** 2
   General procedure for the preparation of alcohol-PA-OTHOs (**9bb-9be)** 3
   Procedure for the preparation of **9ac** 4
   General protocol for the preparation of OTHO gels 4
3. Scanning electron microscopy imaging 5
4. Spectral data for all compounds 6
   N,*N*,*N*-trimethyl-2-((6-oxo-3,4-diphenyl-6-(4-(10-phenylanthracen-9-yl)phenyl)hexanoyl)oxy)ethan-1-aminium iodide (**6ba**) 6
   N,*N*,*N*-trimethyl-2-((6-oxo-3,4-diphenyl-6-(4-(10-phenylanthracen-9-yl)phenyl)hexanoyl)oxy)ethan-1-aminium tetrakis(3,5-bis(trifluoromethyl)phenyl)borate (**6ba-BARF**) 6
   Potassium 2-((6-oxo-3,4-diphenyl-6-(4-(10-phenylanthracen-9-yl)phenyl)hexanoyl)oxy)ethyl sulfate (**9bb**) 6
   Potassium 3-((6-oxo-3,4-diphenyl-6-(4-(10-phenylanthracen-9-yl)phenyl)hexanoyl)oxy)propane-1,2-diyl bis(sulfate) (**9bc**) 7
   Potassium ((2-((6-oxo-3,4-diphenyl-6-(4-(10-phenylanthracen-9-yl)phenyl)hexanoyl)oxy)ethyl)azanediyl)bis(ethane-2,1-diyl) bis(sulfate)) (**9bd**) 7
   Potassium 2-(((6-oxo-3,4-diphenyl-6-(4-(10-phenylanthracen-9-yl)phenyl)hexanoyl)oxy)methyl)-2-((sulfonatooxy)methyl)propane-1,3-diyl bis(sulfate) (**9be**) 7
   Potassium 3-((6-oxo-3,4,6-triphenylhexanoyl)oxy)propane-1,2-diyl bis(sulfate) (**9ac**) 8
5. Copies of NMR spectra 9
6. FTIR analysis of **9bc** and its xerogels 16

1. General information

All reagents and solvents were sourced from Merck, TCI, Techno Fisher Scientific and Acros and were used without additional purification. All moisture-sensitive reactions were performed under nitrogen with standard Schlenk techniques. Dry acetonitrile was retrieved from an SPS PS-MD-5/7 dry solvent system. Ultrapure water was obtained from a Merck MiliQ system. Column chromatography was performed with a Biotage Selekt automatic purification system using Biotage Sfär 10 and 25g Silica 60 cartridges and dichloromethane/methanol mixtures. Analytical thin-layer chromatography (TLC) was carried out on VWR or Merck 60 F254 pre-coated silica gel plates (0.2 mm thickness) and visualized with 254 nm light. All compounds prepared have a purity of at least 95% as measured by NMR. Traces of solvent (EtOH, DCM) have been accounted for when calculating yields.
Proton (^{1}H) and carbon nuclear magnetic resonance spectra (^{13}C NMR) were recorded on Bruker Avance 600 or 800 MHz spectrometers in deuterated chloroform ($CDCl_3$) and dimethylsulfoxide (DMSO-d_6). Spectra were referenced to the residual solvent signals. Chemical shifts (δ) are expressed in parts per million (ppm) and coupling constants (*J*) in hertz. LC-MS analysis was carried out with a Waters Acquity Arc HPLC connected to an Acquity QDa Mass Detector equipped with an electrospray ionization source and an ion-trap detector. High resolution mass spectral analysis (HRMS) was performed on a Thermo Fisher Scientific UltiMate 3000 HPLC system coupled to a Q Exactive HF Orbitrap mass spectrometer equipped with an electrospray ionization source operated in positive or negative mode depending on the nature of the sample. IR spectra of the compounds were performed in neat samples with a Bruker INVENIO FT-IR ATR spectrometer. Peaks (υ) are reported in cm^{-1}.
Rheology was performed on an Anton Paar MCR500 oscillatory rheometer using a PP25 parallel plate probe (Ø = 25 mm, test gap 0.05 mm in all cases except **9ac** gel in 0.1M $CaCl_2$, which uses 0.1 mm). Temperature was kept at a constant 25 ºC using a Peltier device. For amplitude sweeps, strain was measured between 0.01 and 100% or up to 200% for the **9ac** 0.05 M $CaCl_2$ gel at 6.28 rad/s. Frequency sweeps were recorded between 0.01 and 100 rad/s at 0.5% strain. Flow points were calculated using RheoPlus software. SEM images were obtained using a Zeiss Gemini II 450 scanning electron microscope equipped with a Denka Thermal FEG gun. Probe current was set at 200 pA with an accelerating voltage of 1.5 kV. Samples were prepared by placing a drop of gel dispersion (ca. 20 µL of gel in 1 mL of MiliQ water dispersed through 37 kHz ultrasound) on top of a piece of silicon wafer mounted on carbon tape, then drying under vacuum overnight. Image post-processing and fiber measurements were carried out using ImageJ 1.54g. Square objects in the order of µm can be observed in the images and correspond to crystals of the salts used, not the compounds or gels themselves. $T_{gel-sol}$ of the gels was determined using a Büchi B545 melting point apparatus by loading the gel in an open capillary tube. The gel was pushed 2 mm away from the edge of the tube and allowed to set. $T_{gel-sol}$ was considered as the temperature in which the gel started to flow to the bottom of the tube.

2. Gelation and synthetic protocols

Procedure for the preparation of **6ba** and **7ba**

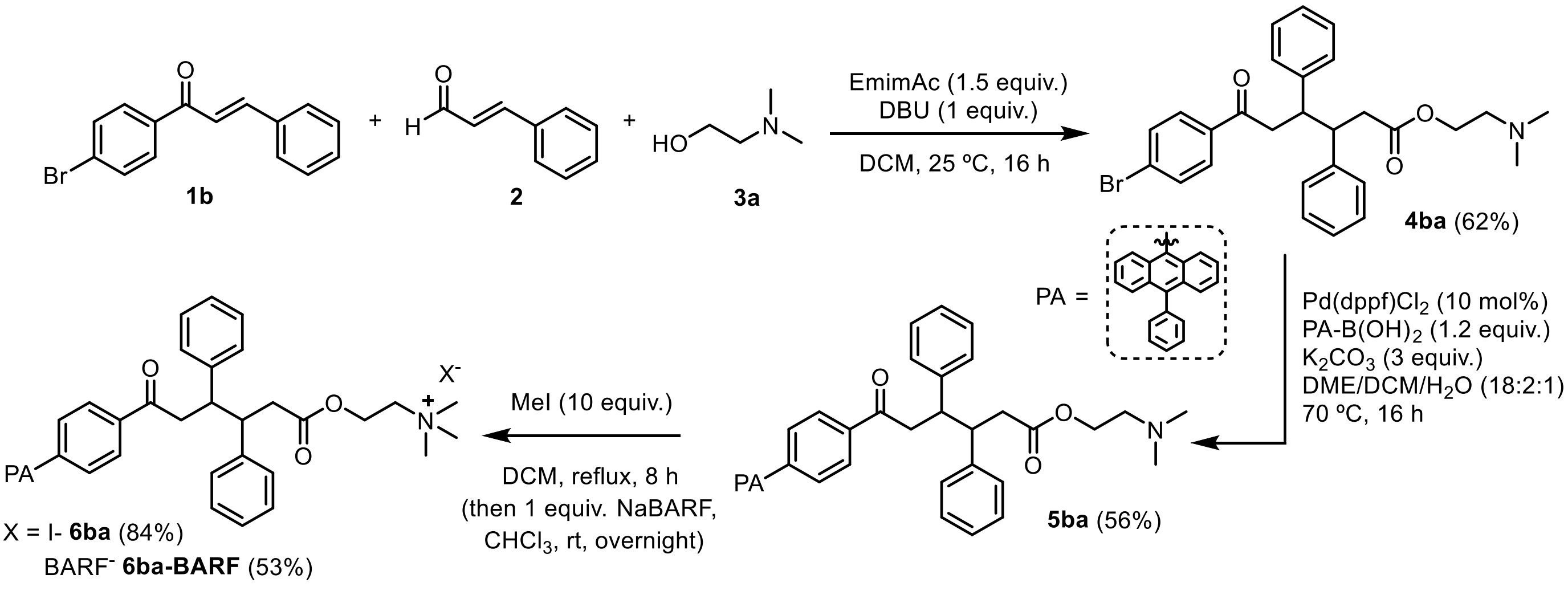

6ba was prepared following reported procedures. In a 50 mL round bottom flask, *(E)*-4'-bromochalcone (574 mg, 2 mmol), cinnamaldehyde (755 µL, 6 mmol, 3 equiv.), EmimAc (516 mg, 3 mmol), *N,N*-dimethylethanolamine (2 mL, 20 mmol) and DBU (300 µL, 2 mmol) were dissolved in dichloromethane (20 mL) and stirred at room temperature for 16 h. Then, the solvent was removed under reduced pressure and cold methanol (10 mL) was added. This slurry was stirred vigorously at 0 ºC until precipitate formed. The resulting solid residue was filtered, washed with cold methanol, and dried, affording 630 mg of **4ba** (62%) as a pure white solid. Then, a Schlenk tube was charged with **4ba** (250 mg, 0.5 mmol), 10-phenyl-9-anthraceneboronic acid (180 mg, 0.6 mmol) and potassium carbonate (207 mg, 1.5 mmol). The components were dissolved in a mixture of 1,2-dimethoxyethane/dichloromethane/water (9/1/0.1 mL) and stirred at room temperature for 30 minutes under inert atmosphere. Then, Pd(dppf)Cl_2 (37 mg, 10 mol%) was added and the mixture was stirred at 70 ºC for 16 h. After this, water was added (10 mL) and the aqueous phase was extracted with dichloromethane (3x20 mL). The combined organic phases were dried over magnesium sulfate and dried. Removing the solvent afforded the crude product, which was then purified by column chromatography (dichloromethane to 95:5 dichloromethane/methanol), yielding 190 mg of **5ba** as a yellow solid. Afterwards, in a 10 mL round bottom flask, **5ba** (100 mg, 0.15 mmol) and methyl iodide (91 µL, 1.5 mmol) were dissolved in 5 mL of dichloromethane. The flask was fitted with a condenser, and the mixture was refluxed for 8 h, triggering the formation of a precipitate, which was filtered and washed with diethyl ether to afford **6ba** as a pure white solid (102 mg, 84%). For the preparation of **7ba**, **6ba** (50 mg, 0.06 mmol) and NaBARF (54 mg, 0.06 mmol) were dissolved in 5 ml of chloroform and stirred at room temperature overnight, causing the formation of precipitate. The mixture was filtered through a thin plug of silica and eluted with dichloromethane. After removing the solvent, **6ba-BARF** was obtained as a vitreous yellow solid (50 mg, 53%).

General procedure for the preparation of alcohol-PA-OTHOs (**9bb-9be)**

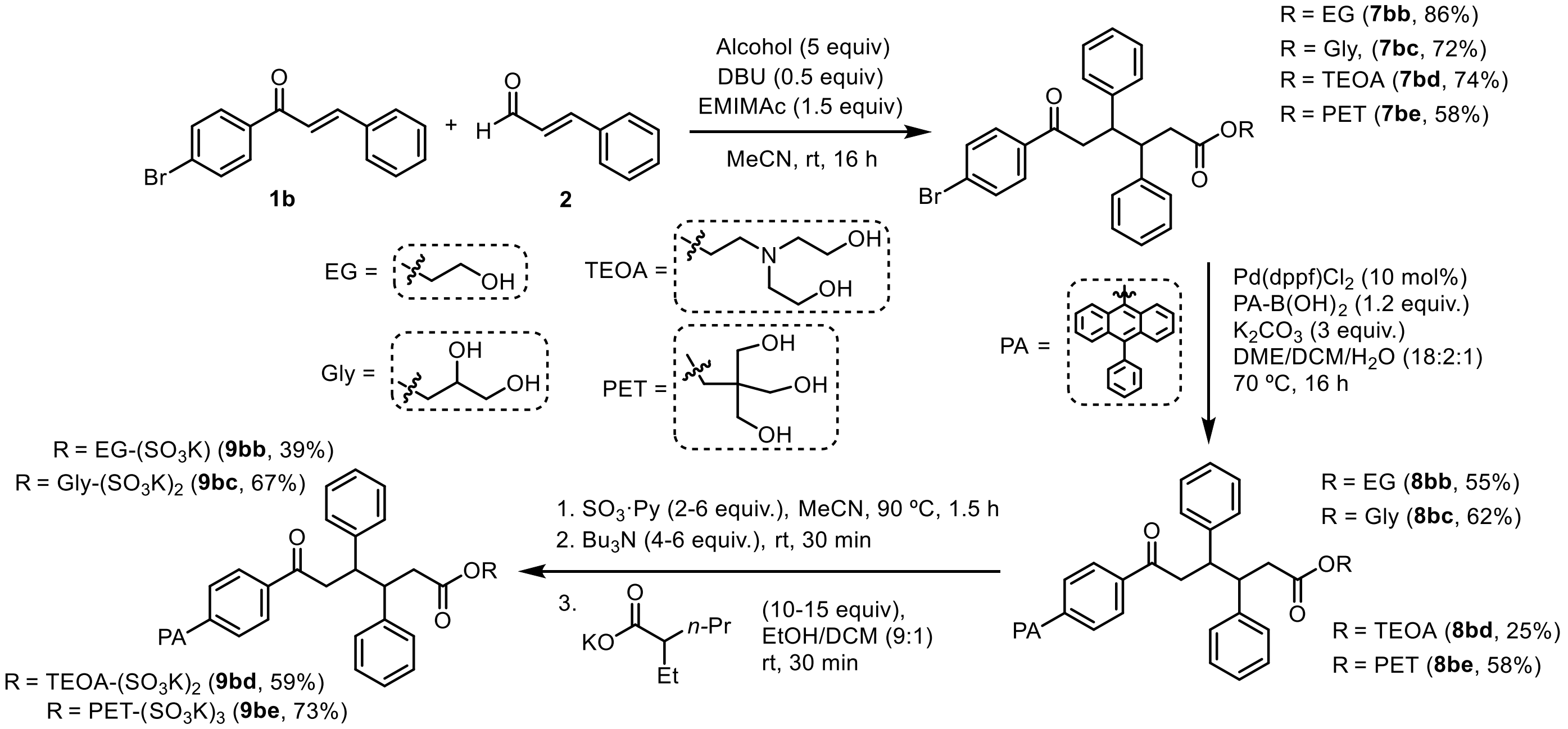

In a round bottom flask, *(E)*-4'-bromochalcone (1 equiv.), cinnamaldehyde (2 equiv.), EmimAc (1.5 equiv.), the corresponding alcohol (5 equiv.) and DBU (0.5 equiv.) were dissolved in acetonitrile (0.15M) and stirred at room temperature for 16 hours. Then, the solvent was removed under reduced pressure and cold methanol (5 mL/mmol) was added. This slurry was stirred vigorously at 0 ºC until precipitate formed. The resulting solid residue was filtered, washed with cold methanol, and dried, affording **7bb-7be** as pure white solids. Then, a Schlenk tube was charged with the compound (1 equiv.), 10-phenyl-9-anthraceneboronic acid (1.2 equiv.) and potassium carbonate (3 equiv.). The components were dissolved in a mixture of 1,2-dimethoxyethane/dichloromethane/water (18:2:1, 0.05M) and stirred at room temperature for 30 minutes under inert atmosphere. Then, Pd(dppf)Cl_2 (10 mol%) was added and the mixture was stirred at 75 ºC for 16 h. After this, water was added and the aqueous phase was extracted with dichloromethane. The combined organic phases were dried over magnesium sulfate and dried. Removing the solvent afforded the crude product, which was then purified by column chromatography (dichloromethane to 95:5 dichloromethane/methanol), yielding **8bb-8be** as yellow solids. For the final step, a round bottom flask was charged with PA-OTHO (1 equiv.) and sulfur trioxide-pyridine complex (2 equiv. per OH), then placed under inert atmosphere. After adding dry acetonitrile (0.1M), the mixture was refluxed for 1.5 hours, confirming full consumption of the starting material by TLC. Then,

tributylamine (2 equiv. per OH) was added. After stirring the mixture for a further 30 minutes at room temperature, the solvent was removed and the residue was purified by column chromatography (dichloromethane to 95:5 dichloromethane/methanol), affording a brownish oil. This oil was redissolved in ethanol/dichloromethane (9:1) and a solution of potassium 2-ethylhexanoate in ethanol (5 equiv. per OSO_3^-, 1M) was slowly dripped into the vessel, causing the immediate precipitation of a solid. After stirring for 20 minutes at room temperature, the solid was vacuum filtered and dried, affording the title compounds as pure white solids.

Procedure for the preparation of **9ac**

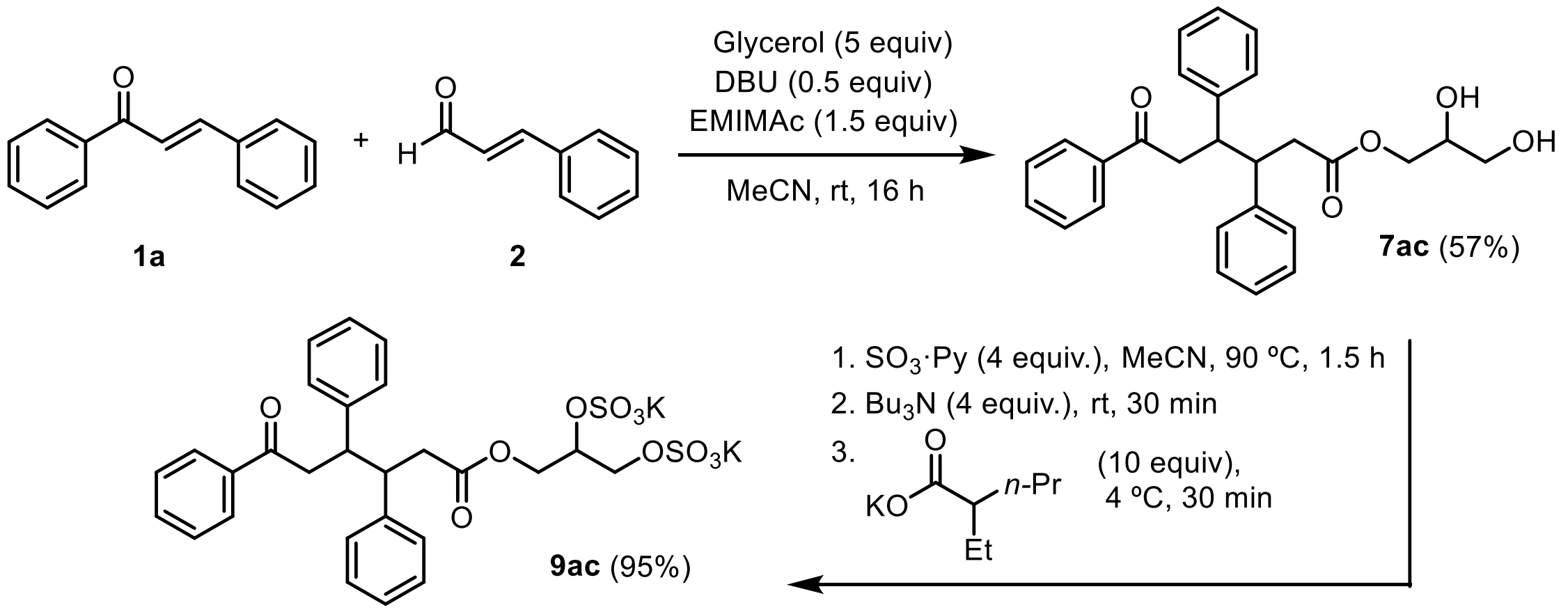

In a round bottom flask, *(E)*-chalcone (1.04 g, 5 mmol), cinnamaldehyde (1.26 mL, 10 mmol, 2 equiv.), EmimAc (1.27 g, 7.5 mmol, 1.5 equiv.), glycerol (1.82 mL, 25 mmol, 5 equiv.) and DBU (0.5 equiv.) were dissolved in acetonitrile (35 mL, 0.13M) and stirred at room temperature for 16 hours. Then, the solvent was removed under reduced pressure and cold methanol (20 mL) was added. This slurry was stirred vigorously at 0 ºC until precipitate formed. The resulting solid residue was filtered, washed with cold methanol, and dried, affording **7ac** as a pure white solid (1.23 g, 2.85 mmol, 57%). For the final step, a round bottom flask was charged with **7ac** (200 mg, 0.45 mmol) and sulfur trioxide-pyridine complex (294 mg, 1.85 mmol, 4 equiv.), then placed under inert atmosphere. After adding dry acetonitrile (20 mL), the mixture was refluxed for 1.5 hours, confirming full consumption of the starting material by TLC. Then, tributylamine (440 µL, 1.85 mmol, 4 equiv.) was added. After stirring the mixture for a further 30 minutes at room temperature, the solvent was removed and the residue was purified by column chromatography (dichloromethane to 95:5 dichloromethane/methanol), affording a brownish oil. This oil was redissolved in ethanol/dichloromethane (9:1) and a solution of potassium 2-ethylhexanoate in ethanol (843 mg, 4.5 mmol, 10 equiv. in 4.5 mL of EtOH) was slowly dripped into the vessel, causing the immediate precipitation of a solid. After stirring for 20 minutes, the solid was vacuum filtered and dried, affording the title compound as a pure white solid (294 mg, 0.44 mmol, 95% yield).

General protocol for the preparation of OTHO gels

The appropriate gelator is weighed into a glass vial, and water was added until the desired concentration was reached. The vial was closed with a screw cap and the mixture heated with a heat-gun until full dissolution of the gelator. For tests involving the presence of ions, an adequate amount of 1M solution of the ion was pipetted directly into the hot gelator solution, then reheated until fully clear and allowed to stand at room temperature. Gelation was assessed through the inverted vial test (IVT).

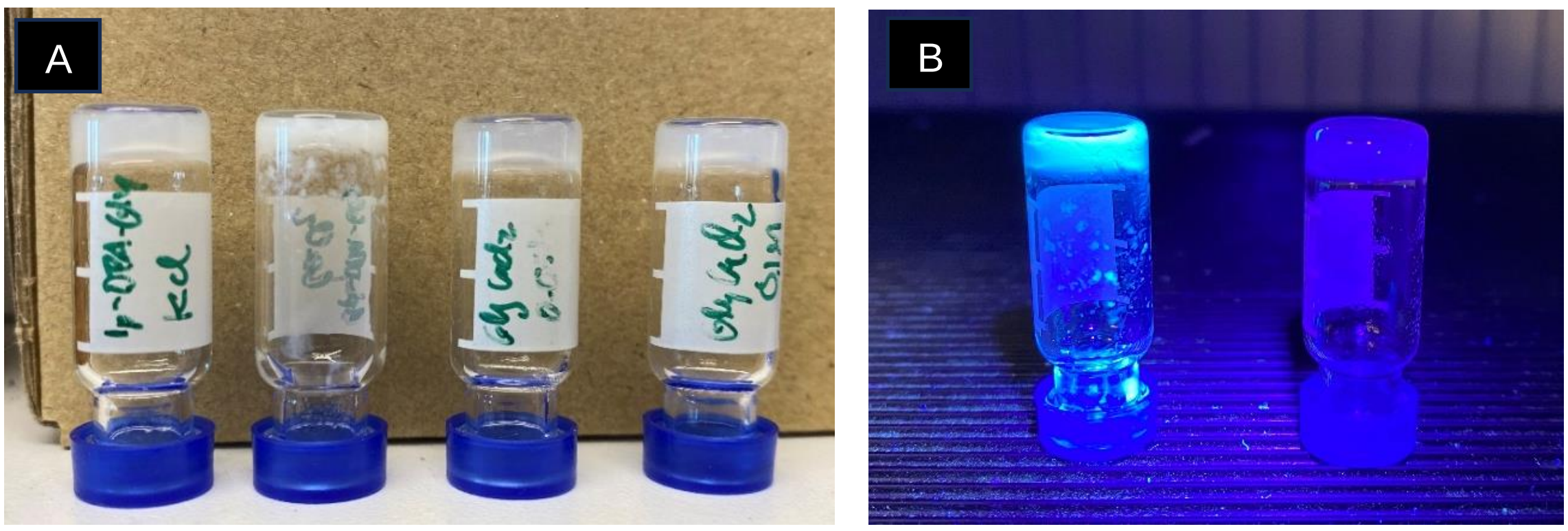

Figure S1. A) Gels prepared for this study. Left to right, **9bc** in 0.05M KCl, **9bc** in 0.05M $CaCl_2$, **9ac** in 0.05M $CaCl_2$ and **9ac** in 0.1M $CaCl_2$. B) **9bc** in 0.05M KCl (left) **9ac** in 0.05M $CaCl_2$ (right) visualized under 365 nm light. Note the absence of fluorescence for the non-PA **9ac**.

3. Scanning electron microscopy imaging

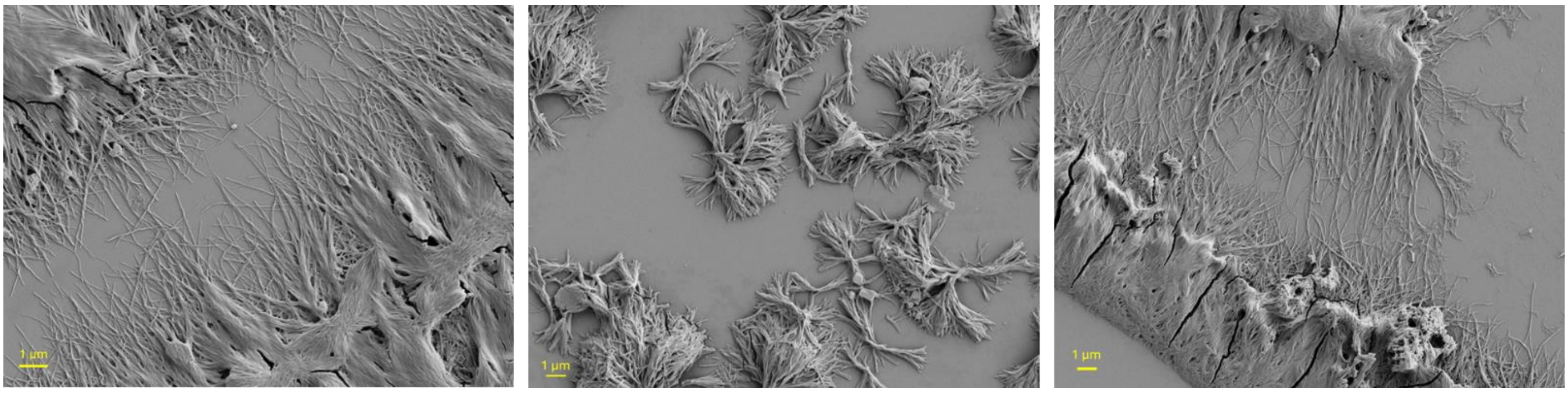

Figure S2. SEM images of the xerogel from **9ac** in 0.1M $CaCl_2$

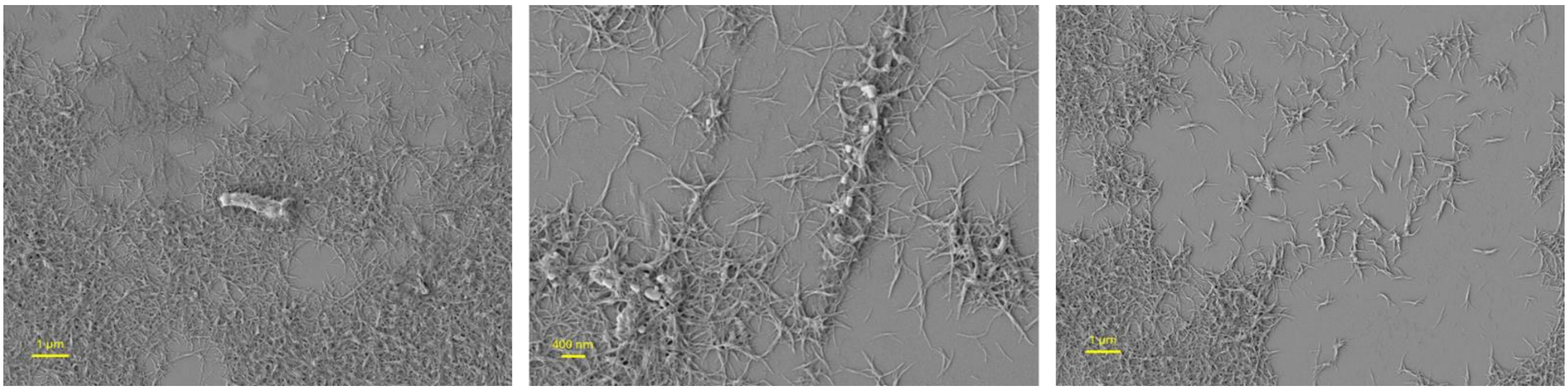

Figure S3. SEM images of the xerogel from **9ac** in 0.05M $CaCl_2$

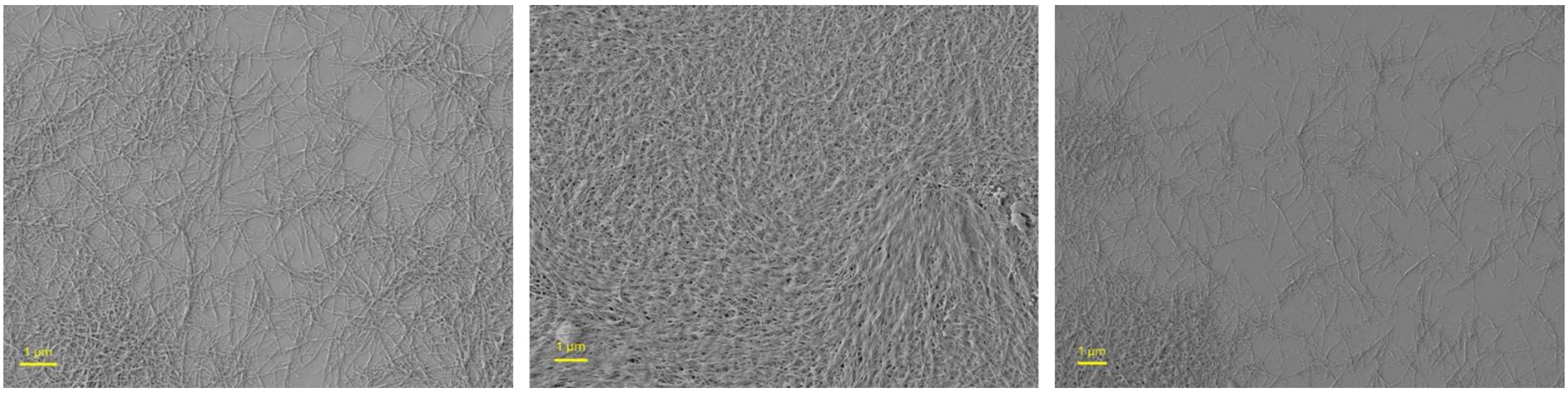

Figure S4. SEM images of the xerogel from **9bc** in 0.05M $CaCl_2$

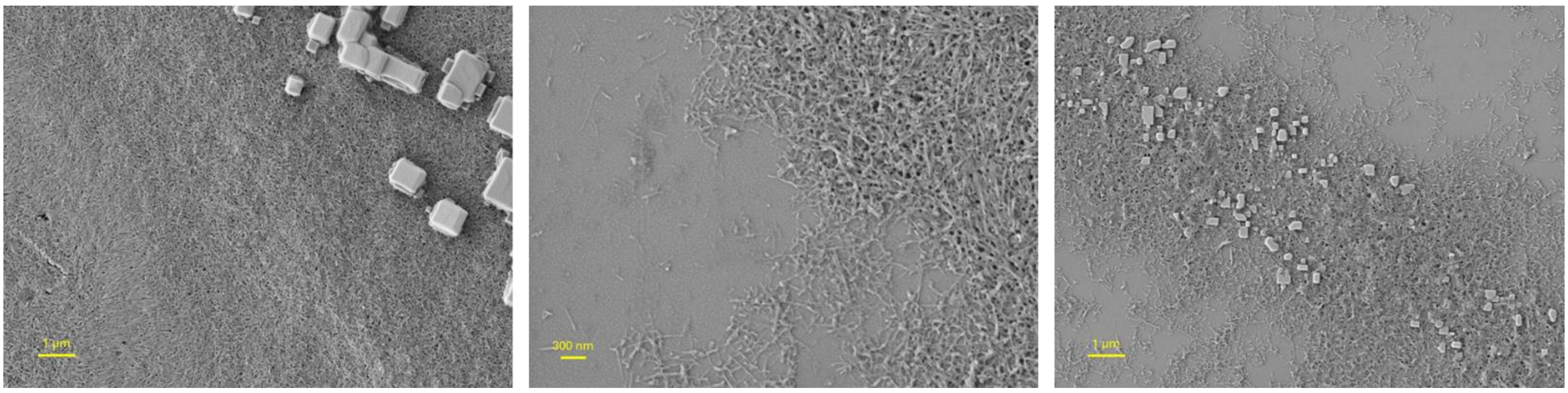

Figure S5. SEM images of the xerogel from **9bc** in 0.05M KCl

4. Spectral data for all compounds

N,*N*,*N*-trimethyl-2-((6-oxo-3,4-diphenyl-6-(4-(10-phenylanthracen-9-yl)phenyl)hexanoyl)oxy)ethan-1-aminium iodide (**6ba**)

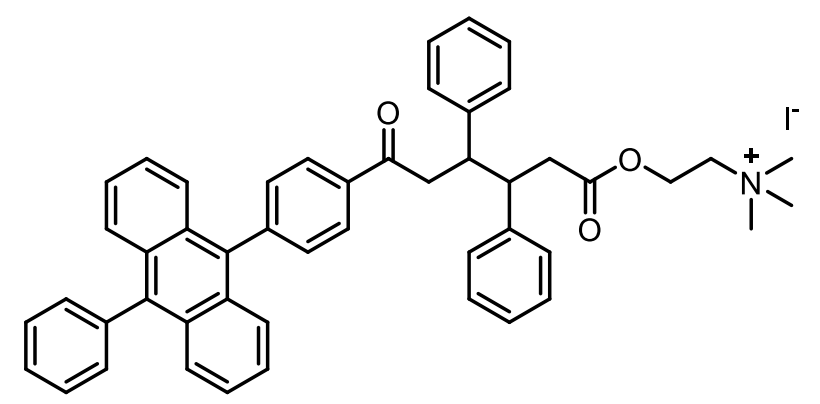

Following the described procedure, compound **6ba** was obtained as a white solid.

^{1}H NMR (600 MHz, DMSO-*d6*) δ_H = 7.98 – 7.93 (m, 2H), 7.68 – 7.64 (m, 2H), 7.63 – 7.59 (m, 1H), 7.58 – 7.56 (m, 2H), 7.52 – 7.46 (m, 6H), 7.46 – 7.32 (m, 11H), 7.28 – 7.21 (m, 2H), 4.18 (br s, 2H), 3.80 – 3.75 (m, 1H), 3.64 (td, *J* = 10.5, 3.5 Hz, 1H), 3.49 – 3.35 (m, 4H), 2.97 (s, 9H), 2.86 – 2.83 (m, 1H), 2.69 (dd, *J* = 15.7, 11.1 Hz, 1H), 2.38 (dd, *J* = 15.7, 3.9 Hz, 1H).; **^{13}C NMR (150 MHz, DMSO-*d6*)** δ_C = 198.3, 171.1, 143.2, 142.9, 142.4, 137.9, 137.1, 135.9, 135.3, 131.3, 130.8, 129.1, 128.8, 128.7, 128.6, 128.5, 128.5, 128.4, 128.1, 127.9, 126.9, 126.6, 126.5, 126.1, 125.8, 125.6, 63.5, 57.6, 52.8, 47.3, 46.5, 42.7, 38.7.; **IR (ATR):** υ = 1725, 1681, 1602, 1369, 1330, 1256, 1225, 1139, 1027, 987, 942, 765, 744, 696, 663, 610, 579, 554, 518, 402 cm^{-1}.; **HRMS (ESI+):** *m/z* calcd. for $C_{49}H_{46}NO_3^+$ 696.3472, observed 696.3477.

N,*N*,*N*-trimethyl-2-((6-oxo-3,4-diphenyl-6-(4-(10-phenylanthracen-9-yl)phenyl)hexanoyl)oxy)ethan-1-aminium tetrakis(3,5-bis(trifluoromethyl)phenyl)borate (**6ba-BARF**)

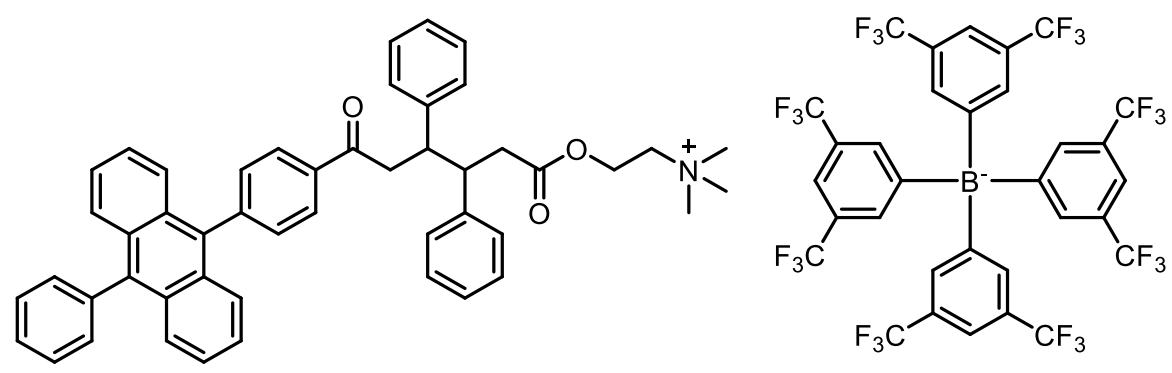

Following the described procedure, compound **6ba-BARF** was obtained as a vitreous yellow solid.
^{1}H NMR (600 MHz, CDCl$_3$) δ_H = 7.80 (br d, *J* = 8.3 Hz, 2H), 7.63 – 7.59 (m, 10H), 7.52 – 7.46 (m, 7H), 7.41 – 7.34 (m, 10H), 7.28 – 7.26 (m, 4H), 7.25 – 7.15 (m, 7H), 4.24 – 4.20 (m, 1H), 3.88 – 3.84 (m, 1H), 3.72 – 3.68 (m, 1H), 3.46 – 3.38 (m, 2H), 2.99 – 2.84 (m, 3H), 2.50 (s, 9H), 2.44 – 2.43 (m, 2H).; **^{13}C NMR (150 MHz, CDCl$_3$)** δ_C = 198.1, 171.5, 161.8 (m), 144.7, 142.2, 142.1, 138.9, 138.0, 136.1, 135.5, 134.9, 131.8, 131.3, 129.9, 129.5, 129.4, 129.3, 129.2, 129.1, 129.0, 128.8, 128.7, 128.6, 128.5, 128.1, 127.8, 127.8, 127.5, 127.4, 127.3, 126.4, 125.6, 125.5, 125.2, 123.8, 121.9, 117.7, 65.9, 56.2, 54.3, 48.4, 46.4, 43.7, 40.2.; **IR (ATR):** υ = 1748, 1678, 1604, 1353, 1273, 1112, 1029, 942, , 885, 838, 761, 701, 681, 667, 402 cm^{-1}.; **HRMS (ESI+):** *m/z* calcd. for $C_{49}H_{46}NO_3^+$ 696.3472, observed 696.3476. **(ESI-):** *m/z* calcd. for $C_{32}H_{12}BF_{24}^-$ 863.0654, observed 863.0636

Potassium 2-((6-oxo-3,4-diphenyl-6-(4-(10-phenylanthracen-9-yl)phenyl)hexanoyl)oxy)ethyl sulfate (**9bb**)

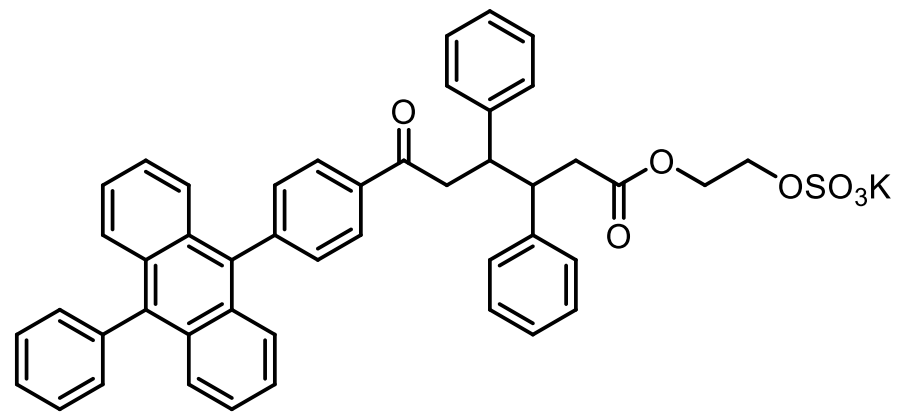

Following the general procedure for the preparation of alcohol-PA-OTHOs using ethylene glycol (**3b**), compound **9bb** was obtained as a white solid.

^{1}H NMR (800 MHz, DMSO-*d6*) δ_H = 7.98 (br d, *J* = 7.7 Hz, 2H), 7.67 – 7.65 (m, 2H), 7.61 – 7.59 (m, 1H), 7.57 (br d, *J* = 8.3 Hz, 2H), 7.50 (br d, *J* = 7.7 Hz, 2H), 7.47 – 7.44 (m, 8H), 7.42 – 7.39 (m, 4H), 7.36 – 7.33 (m, 4H), 7.24 – 7.21 (m, 2H), 3.92 – 3.89 (m, 1H), 3.82 – 3.78 (m, 2H), 3.70 – 3.67 (m, 2H), 3.65 – 3.62 (m, 1H), 3.43 – 3.38 (m, 2H), 2.89 – 2.86 (m, 1H), 2.66 – 2.62 (m, 1H), 2.34 – 2.32 (m, 2H).; **^{13}C NMR (200 MHz, DMSO-*d6*)** δ_C = 198.3, 171.4, 143.1, 142.8, 142.4, 138.0, 137.1, 135.9, 135.4, 131.2, 130.9, 129.1, 128.8, 128.7, 128.5, 128.4, 128.4, 128.3, 128.1, 127.8, 126.7, 126.6, 126.4, 126.1, 125.8, 125.6, 63.4, 62.8, 47.3, 46.5, 42.5, 38.7.; **IR (ATR):** υ = 1718, 1686, 1585, 1453, 1397, 1221, 1151, 1071, 1028, 1008, 987, 917, 828, 799, 773, 761, 700, 617, 581, 562, 518 cm^{-1}.; **HRMS (ESI-):** *m/z* calcd. for $C_{46}H_{37}O_7S^-$ 733.2265, observed 733.2265.

Potassium 3-((6-oxo-3,4-diphenyl-6-(4-(10-phenylanthracen-9-yl)phenyl)hexanoyl)oxy)propane-1,2-diyl bis(sulfate) (**9bc**)

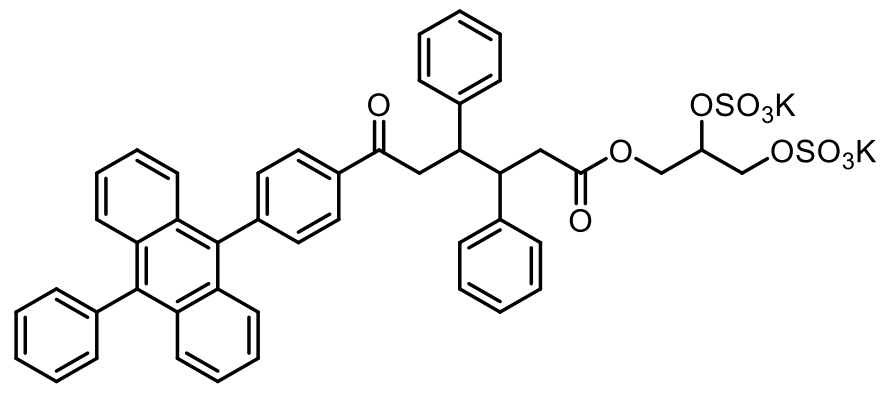

Following the general procedure for the preparation of alcohol-PA-OTHOs using glycerol (**3c**), compound **9bc** was obtained as a white solid.

^{1}H NMR (600 MHz, DMSO-*d6*) δ_H = 7.98 – 7.97 (m, 2H), 7.67 – 7.65 (m, 2H), 7.62 – 7.59 (m, 1H), 7.59 – 7.56 (m, 2H), 7.51 – 7.49 (m, 2H), 7.47 – 7.43 (m, 7H), 7.41 – 7.38 (m, 4H), 7.36 – 7.32 (m, 4H) 7.25 – 7.22 (m, 2H), 4.73 (dd, *J* = 5.2, 3.6 Hz, 1H), 4.53 – 4.50 (m, 1H), 3.81 – 3.71 (m, 2H), 3.62 – 3.59 (m, 1H), 3.47 – 3.41 (m, 1H), 3.24 – 3.17 (m, 2H), 2.89 – 2.85 (m, 1H), 2.66 – 2.60 (m, 1H), 2.35 – 2.30 (m, 1H).; **^{13}C NMR (150 MHz, DMSO-*d6*)** δ_C = 198.3, 171.5, 143.1, 142.9, 142.5, 138.0, 137.1, 135.9, 135.4, 131.2, 130.8, 129.1, 128.8, 128.7, 128.5, 128.4, 128.3, 128.3, 128.2, 128.0, 127.8, 126.7, 126.5, 126.4, 126.1, 125.8, 125.6, 69.1, 69.1, 65.4, 65.3, 62.6, 62.5, 47.2, 47.2, 46.4, 42.6, 38.7, 38.6.; **IR (ATR):** υ = 1731, 1679, 1226, 1155, 1012, 942, 759, 699, 560 cm^{-1}.; **HRMS (ESI-):** *m/z* calcd. for $C_{47}H_{38}O_{11}S_2^{2-}$ 421.0934, observed 421.0931.

Potassium ((2-((6-oxo-3,4-diphenyl-6-(4-(10-phenylanthracen-9-yl)phenyl)hexanoyl)oxy)ethyl)azanediyl)bis(ethane-2,1-diyl) bis(sulfate)) (**9bd**)

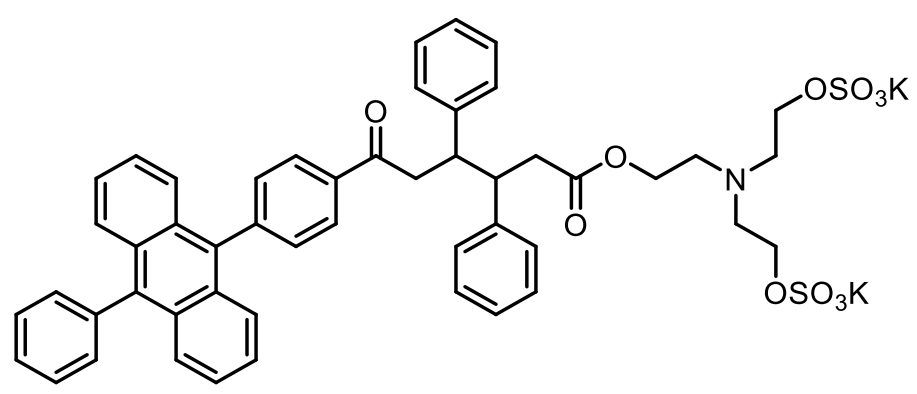

Following the general procedure for the preparation of alcohol-PA-OTHOs using triethanolamine (**3d**), compound **9bd** was obtained as a white solid.

^{1}H NMR (600 MHz, DMSO-*d6*) δ_H = 8.00 – 7.98 (m, 2H), 7.67 – 7.65 (m, 2H), 7.62 – 7.56 (m, 3H), 7.50 – 7.42 (m, 10H), 7.42 – 7.37 (m, 3H), 7.36 – 7.31 (m, 4H), 7.26 – 7.21 (m, 2H), 4.34 (t, *J* = 5.2 Hz, 1H), 4.25 (t, *J* = 5.2 Hz, 1 H), 3.84 – 3.79 (m, 1H), 3.74 – 3.69 (m, 4H), 3.61 (td, *J* = 10.5, 3.5 Hz, 1H), 3.47 – 3.45 (m, 1H), 3.35 – 3.32 (m, 1H), 2.86 (dd, *J* = 17.2, 3.5 Hz, 1H), 2.61 – 2.58 (m, 3H), 2.48 – 2.46 (m, 3H), 2.29 (dd, *J* = 15.3, 4.1 Hz, 1H).; **^{13}C NMR (150 MHz, DMSO-*d6*)** δ_C = 198.4, 171.4, 143.1, 142.8, 142.4, 138.0, 137.1, 135.9, 135.4, 131.2, 130.9, 129.1, 128.8, 128.7, 128.6, 128.5, 128.4, 128.3, 128.3, 128.2, 128.1, 127.8, 126.8, 126.5, 126.4, 126.1, 125.8, 125.6, 63.9, 62.1, 53.4, 52.3, 47.5, 46.6, 42.6.; **IR (ATR):** υ = 1067, 759, 699, 663, 611, 564 cm^{-1}.; **HRMS (ESI-):** *m/z* calcd. for $C_{50}H_{45}NO_{11}S_2^{2-}$ 449.6222, observed 449.6217.

Potassium 2-(((6-oxo-3,4-diphenyl-6-(4-(10-phenylanthracen-9-yl)phenyl)hexanoyl)oxy)methyl)-2-((sulfonatooxy)methyl)propane-1,3-diyl bis(sulfate) (**9be**)

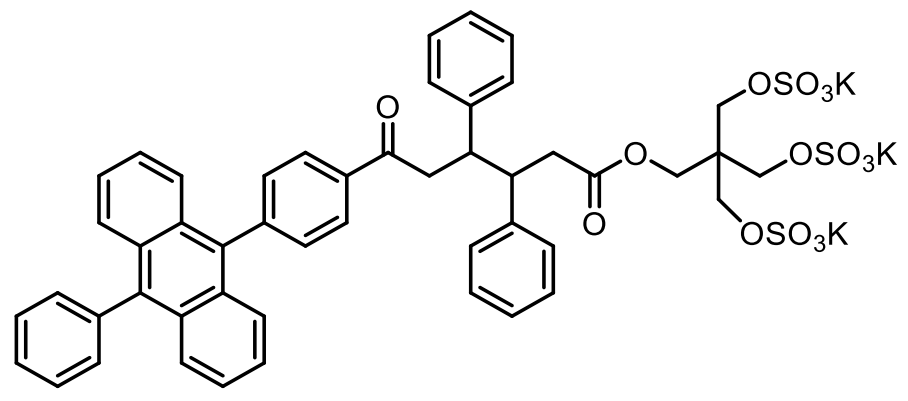

Following the general procedure for the preparation of alcohol-PA-OTHOs using pentaerythritol (**3e**), compound **9be** was obtained as a white solid.

^{1}H NMR (600 MHz, DMSO-*d6*) δ_H = 7.99 – 7.98 (m, 2H), 7.67 – 7.65 (m, 2H), 7.62 – 7.56 (m, 3H), 7.50 – 7.48 (m, 2H), 7.47 – 7.45 (m, 8H), 7.42 – 7.40 (m, 4H), 7.38 – 7.33 (m, 4H), 7.24 – 7.20 (m, 2H), 3.81 – 3.77 (m, 1H), 3.72 – 3.58 (m, 9H), 3.53 (dt, *J* = 10.4, 5.3 Hz, 1H), 2.89 (dd, *J* = 17.1, 3.4 Hz, 1H), 2.58 – 2.54 (m, 1H), 2.35 (dd, *J* = 15.2, 4.2 Hz, 1H).; **^{13}C NMR (150 MHz, DMSO-*d6*)** δ_C = 198.4, 171.3, 143.1, 142.6, 142.4, 138.0, 137.1, 136.1, 135.4, 131.2, 130.9, 129.1, 128.8, 128.7, 128.6, 128.5, 128.4, 128.1, 127.8, 126.7, 126.5, 126.4, 126.1, 125.8, 125.6, 64.8, 63.3, 47.2, 46.8, 42.8, 41.5, 38.9.; **IR (ATR):** υ = 2980, 1684, 1392, 1225, 1069, 988, 814, 772, 704, 665, 581, 479 cm^{-1}.; **HRMS (ESI-):** *m/z* calcd. for $C_{49}H_{42}O_{15}S_3^{2-}$ [M+H] 483.0849, observed 483.0844.

Potassium 3-((6-oxo-3,4,6-triphenylhexanoyl)oxy)propane-1,2-diyl bis(sulfate) (**9ac**)

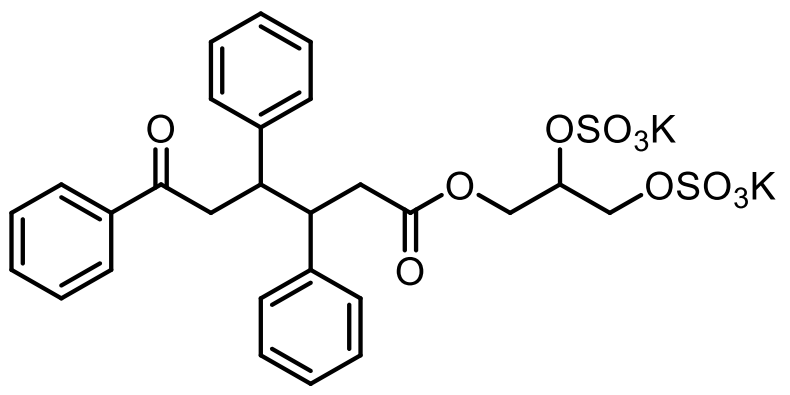

Following the described procedure, compound **9ac** was obtained as a white solid.

^{1}H NMR (600 MHz, DMSO-*d6*) δ_H = 7.69 – 7.97 (m, 2H), 7.54 – 7.52 (m, 1H), 7.41 – 7.39 (m, 4H), 7.35 – 7.30 (m, 4H), 7.27 – 7.25 (m, 2H), 7.21 – 7.18 (m, 1H), 7.16 – 7.14 (m, 1H), 4.27 – 4.22 (m, 1H), 3.91 – 3.80 (m, 2H), 3.76 – 3.64 (m, 2H), 3.57 – 3.51 (m, 2H), 3.44 – 3.42 (m, 1H), 2.75 – 2.71 (m, 1H), 2.54 – 2.52 (m, 1H), 2.31 – 2.27 (m, 1H).; **^{13}C NMR (150 MHz, DMSO-*d6*)** δ_C = 198.6, 171.2, 171.2, 142.5, 142.4, 136.6, 132.9, 128.6, 128.4, 128.4, 128.3, 128.2, 127.7, 126.7, 126.5, 71.6, 71.5, 64.4, 64.2, 62.9, 62.8, 61.2, 47.0, 46.9, 46.6, 46.5, 42.5, 38.7, 38.6.; **IR (ATR):** υ = 1730, 1679, 1227, 1122, 1015, 943, 759, 699, 617, 580 cm^{-1}.; **HRMS (ESI-):** *m/z* calcd. for $C_{27}H_{26}O_{11}S_2^{2-}$ 295.0464, observed 295.0460.

5. Copies of NMR spectra

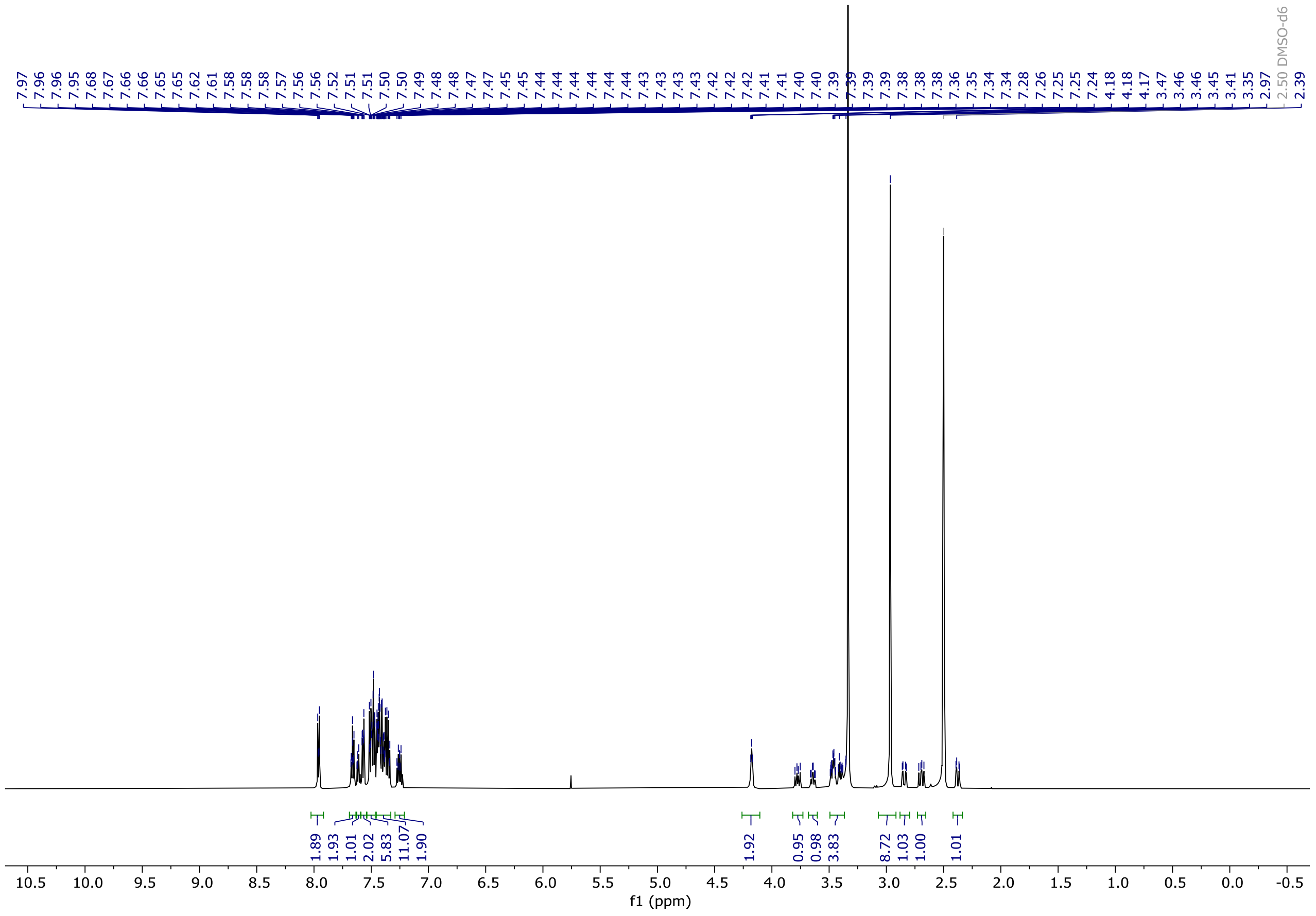

Figure S6. ^{1}H NMR spectrum of **6ba** in DMSO-d_6

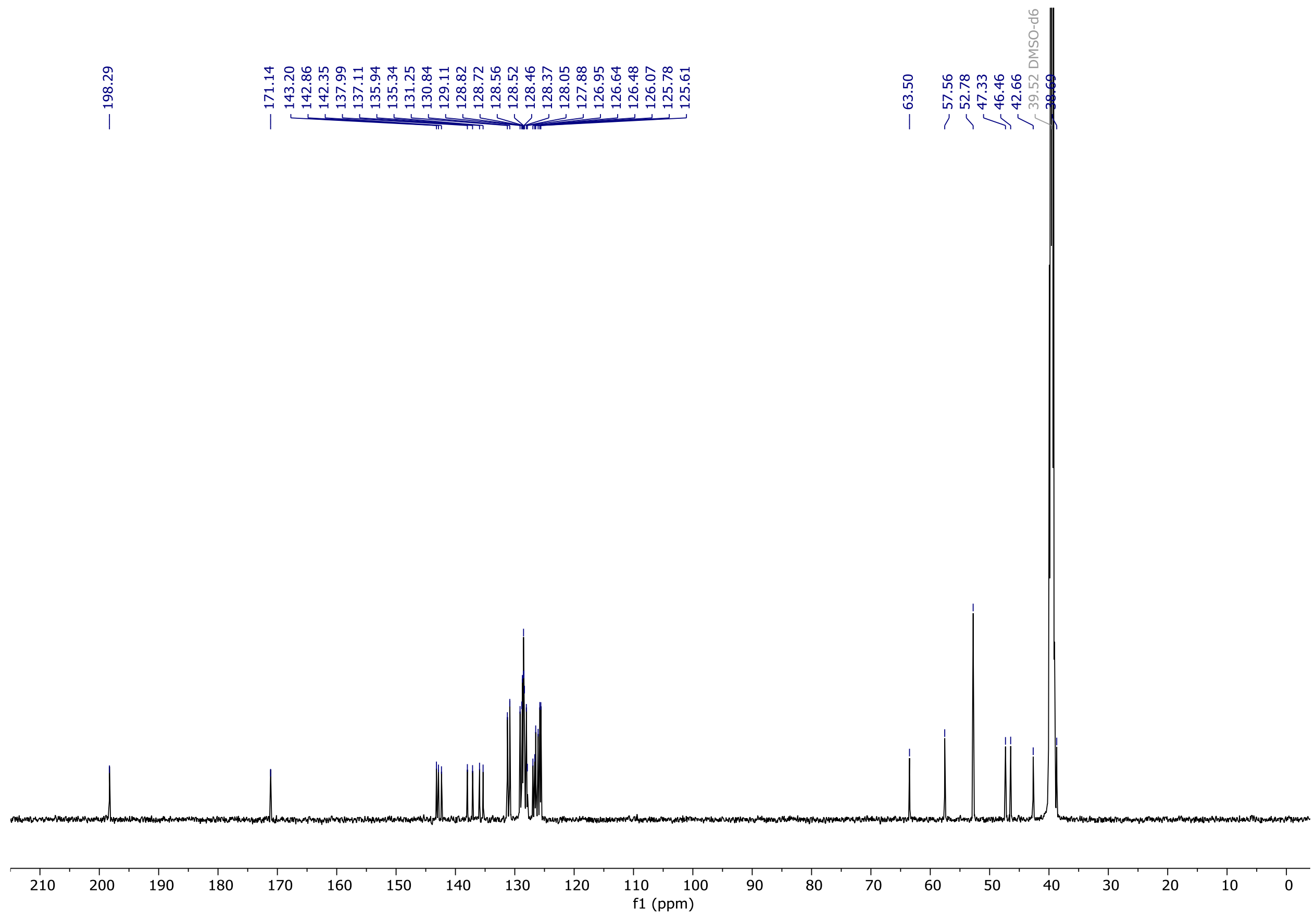

Figure S7. ^{13}C NMR spectrum of **6ba** in DMSO-d_6

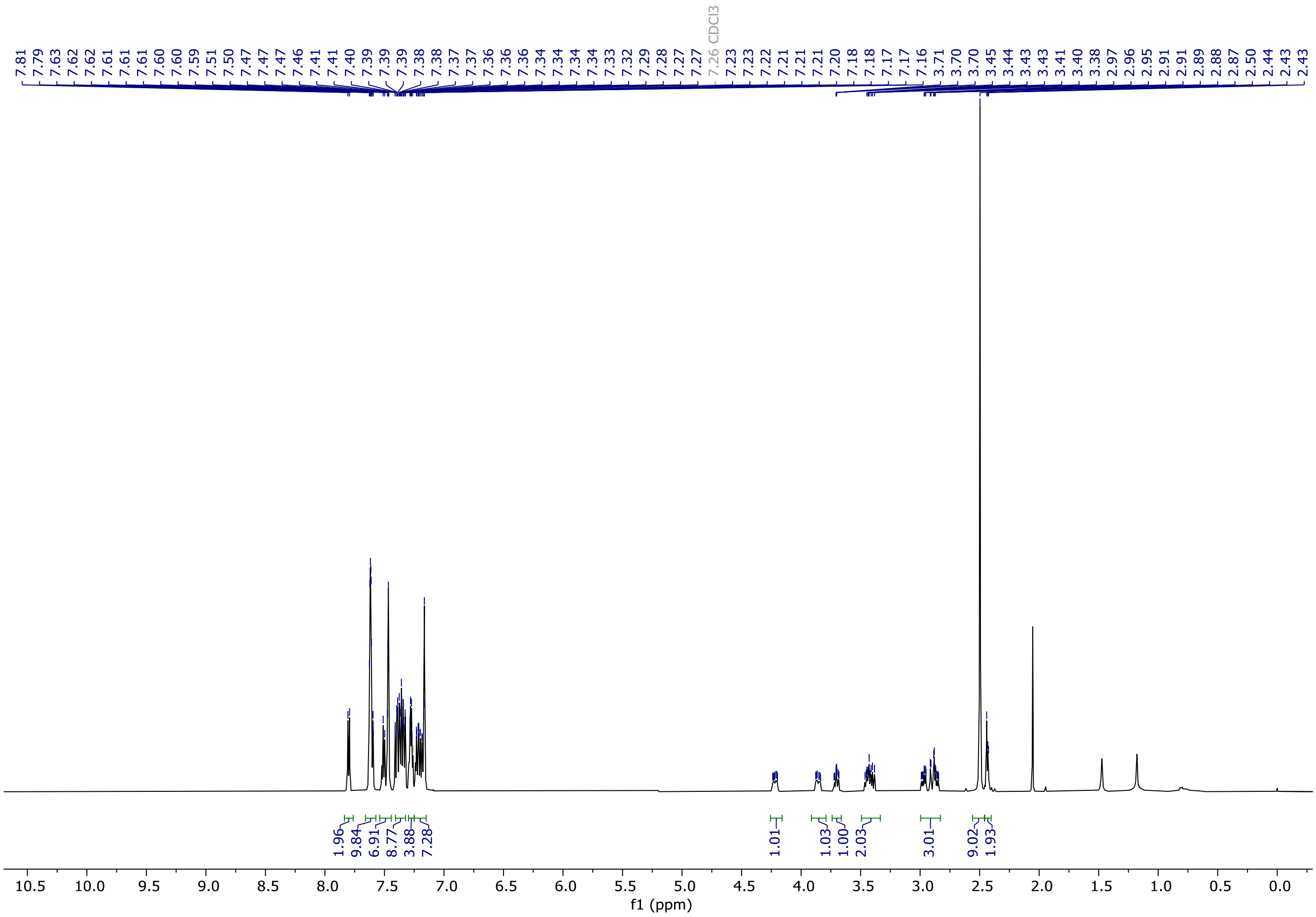

Figure S8. ^{1}H NMR spectrum of **6ba-BARF** in $CDCl_3$

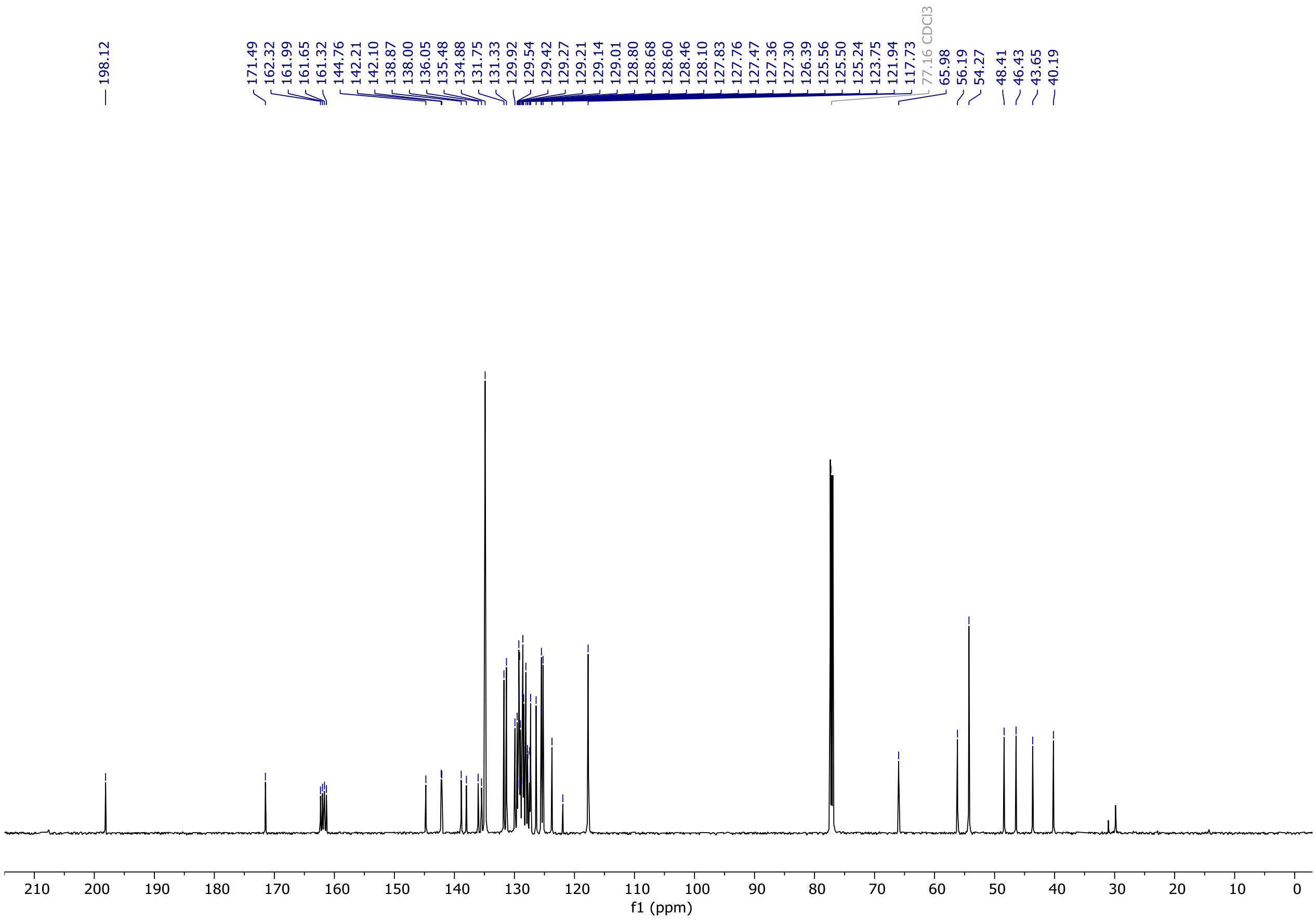

Figure S9. ^{13}C NMR spectrum of **6ba-BARF** in $CDCl_3$

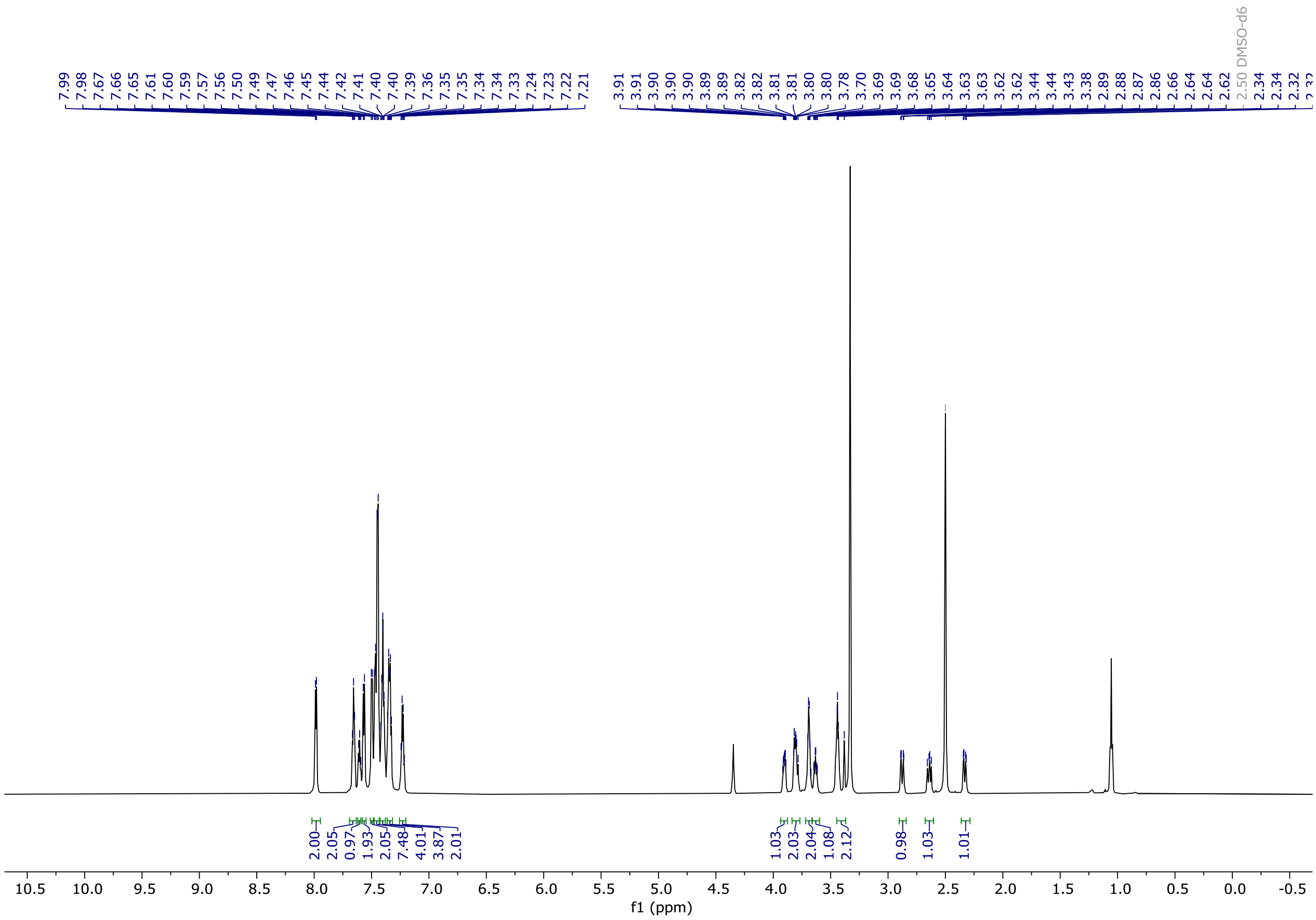

Figure S10. ^{1}H NMR spectrum of **9bb** in DMSO-d_6

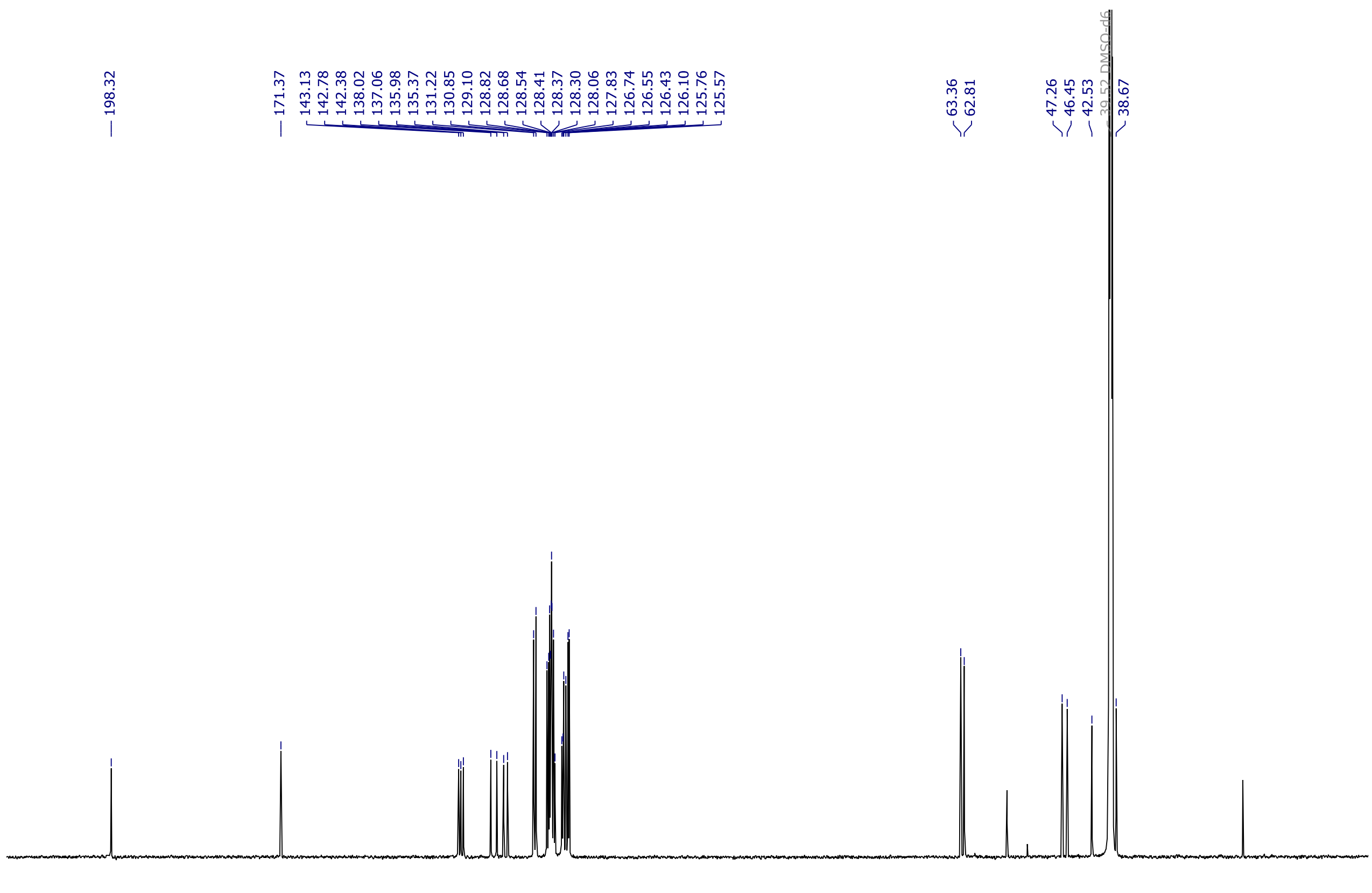

Figure S11. ^{13}C NMR spectrum of **9bb** in DMSO-d_6

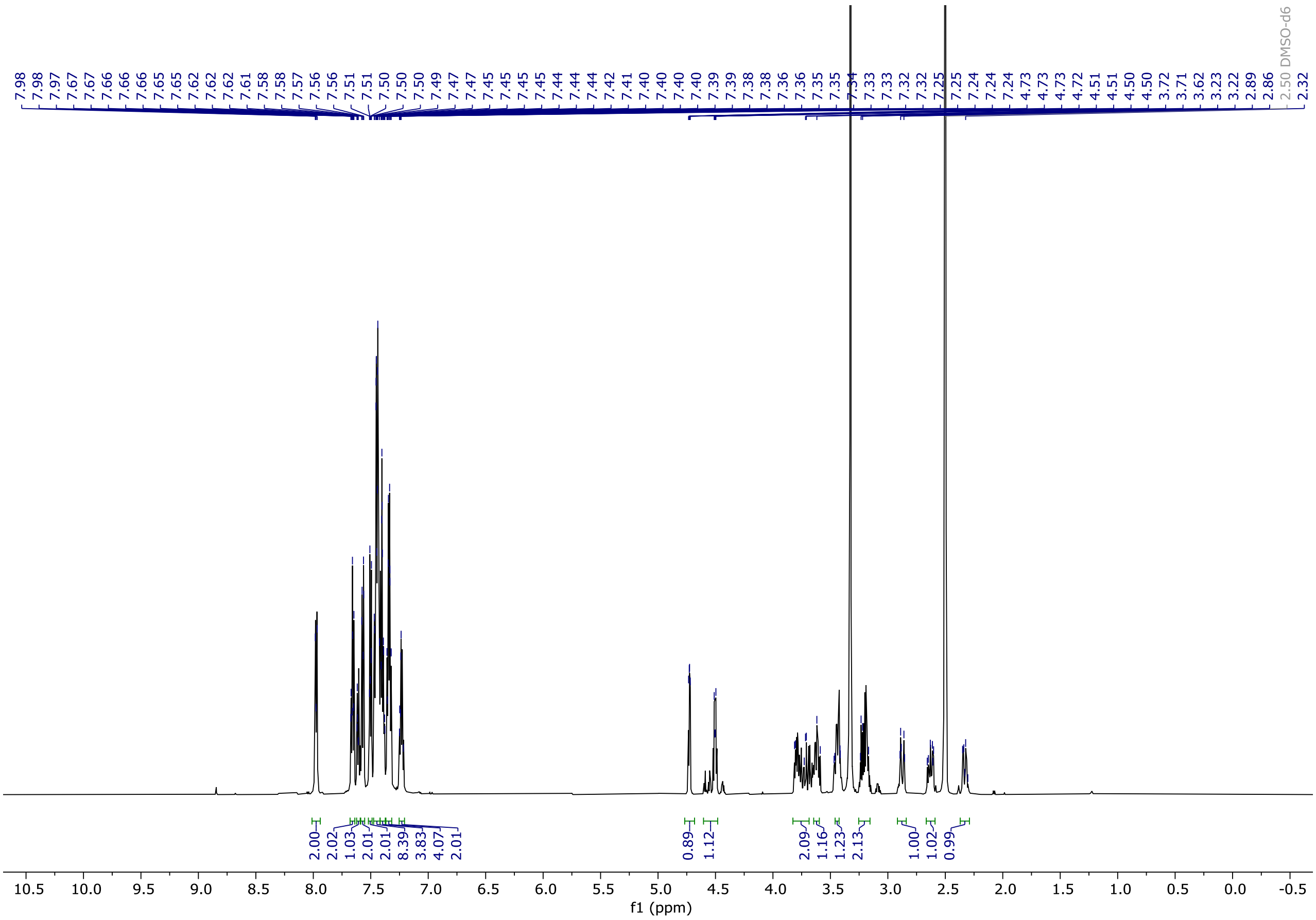

Figure S12. ^{1}H NMR spectrum of **9bc** in DMSO-d_6

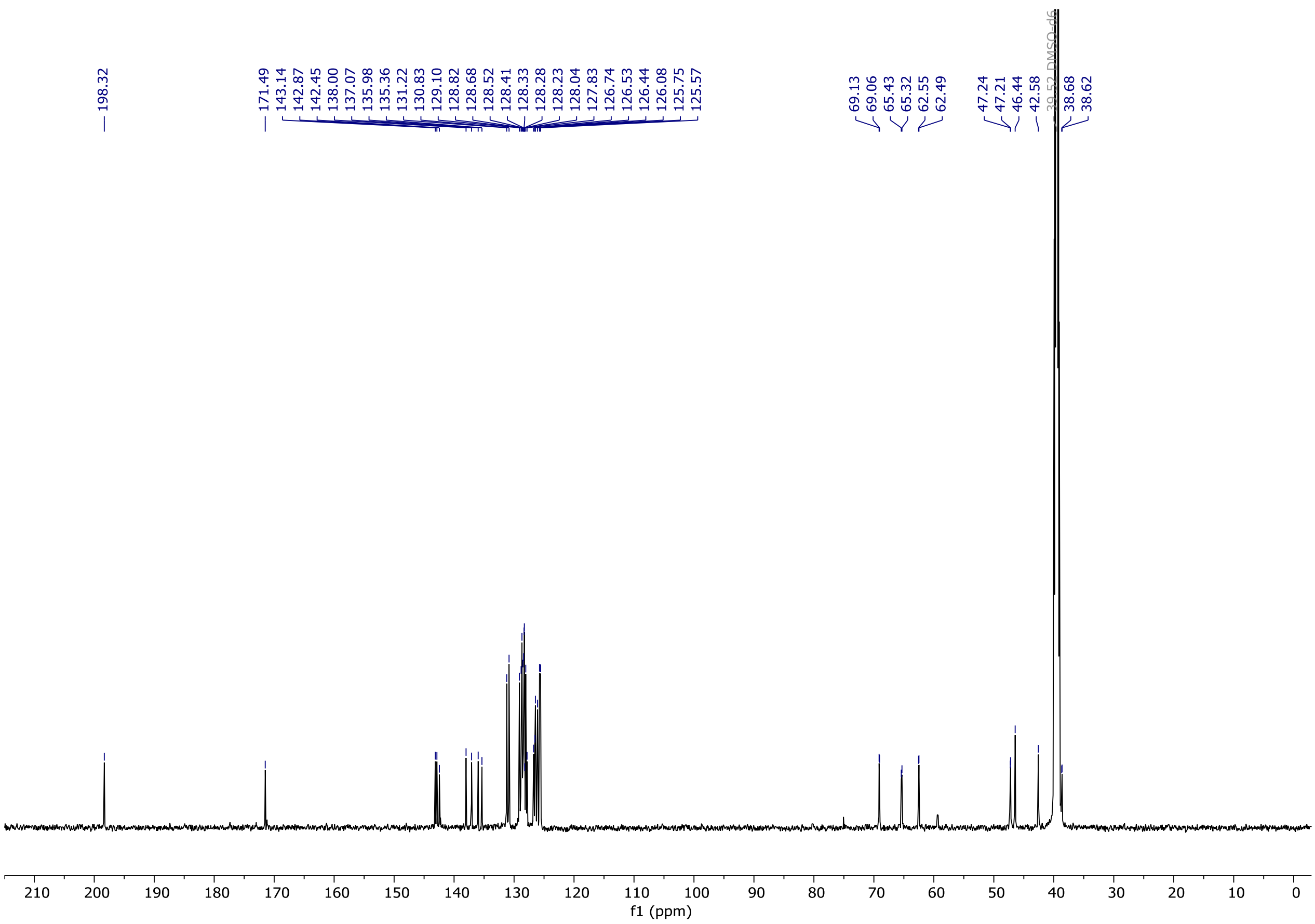

Figure S13. ^{13}C NMR spectrum of **9bc** in DMSO-d_6

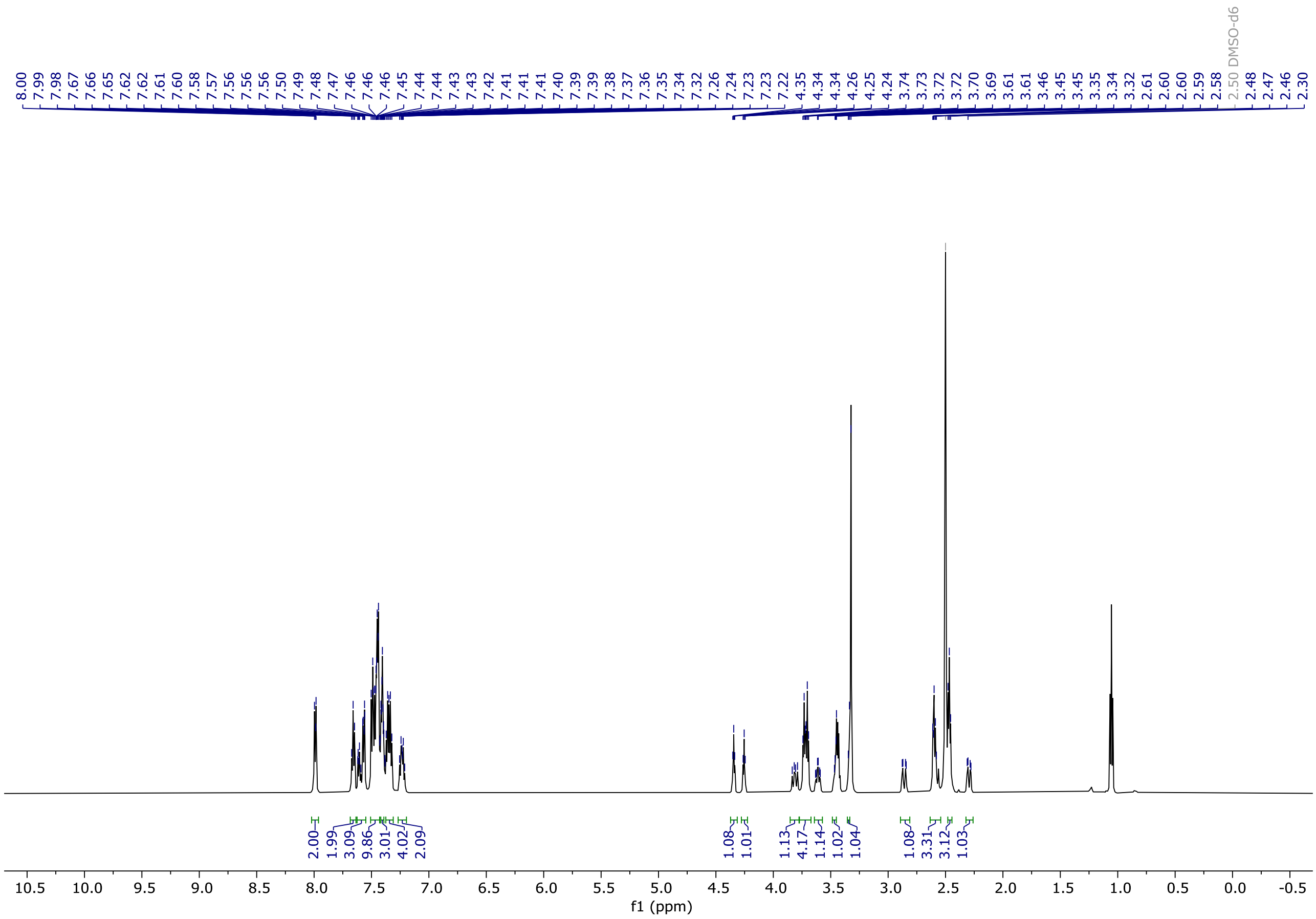

Figure S14. ^{1}H NMR spectrum of **9bd** in DMSO-d_6

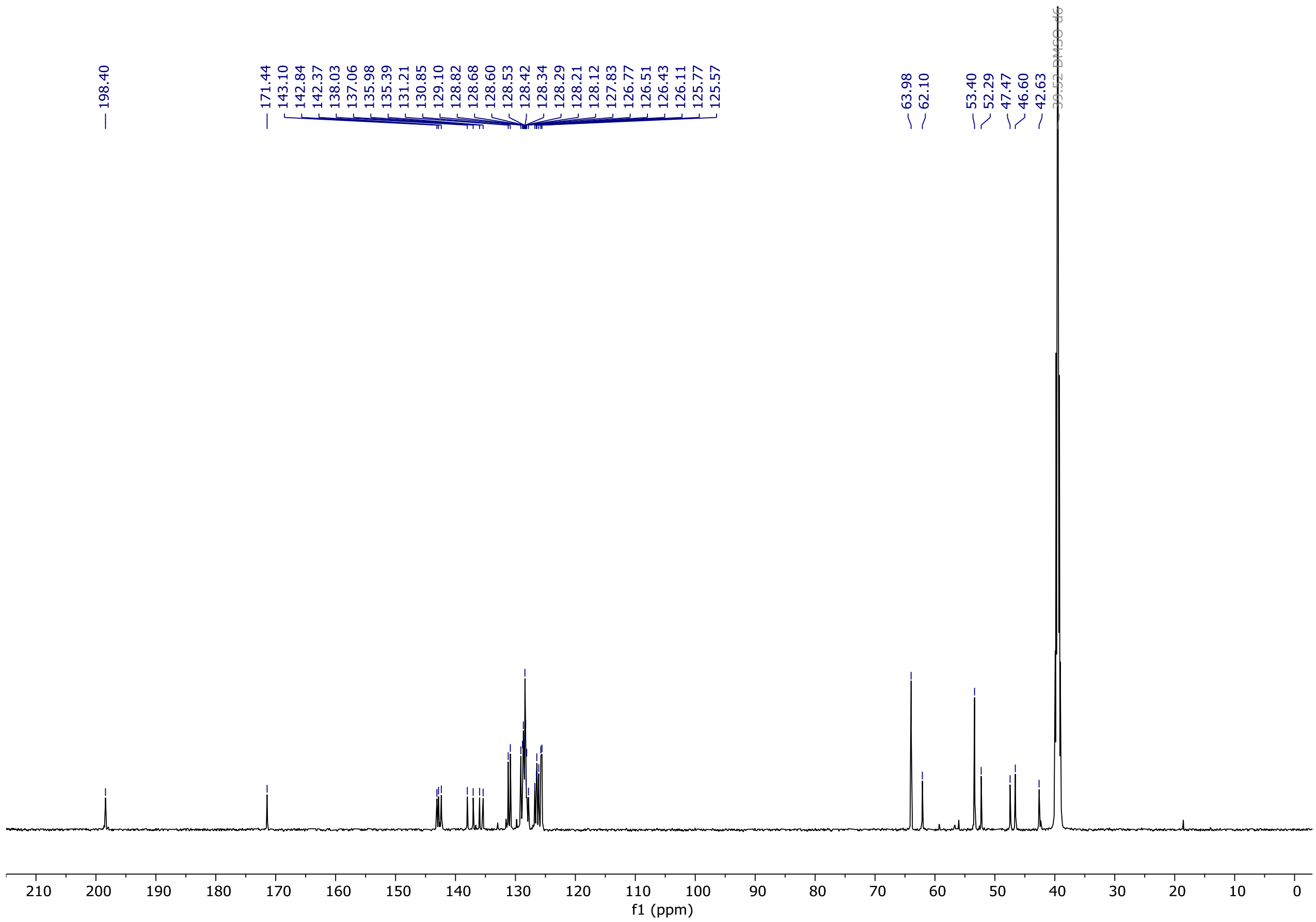

Figure S15. ^{13}C NMR spectrum of **9bd** in DMSO-d_6

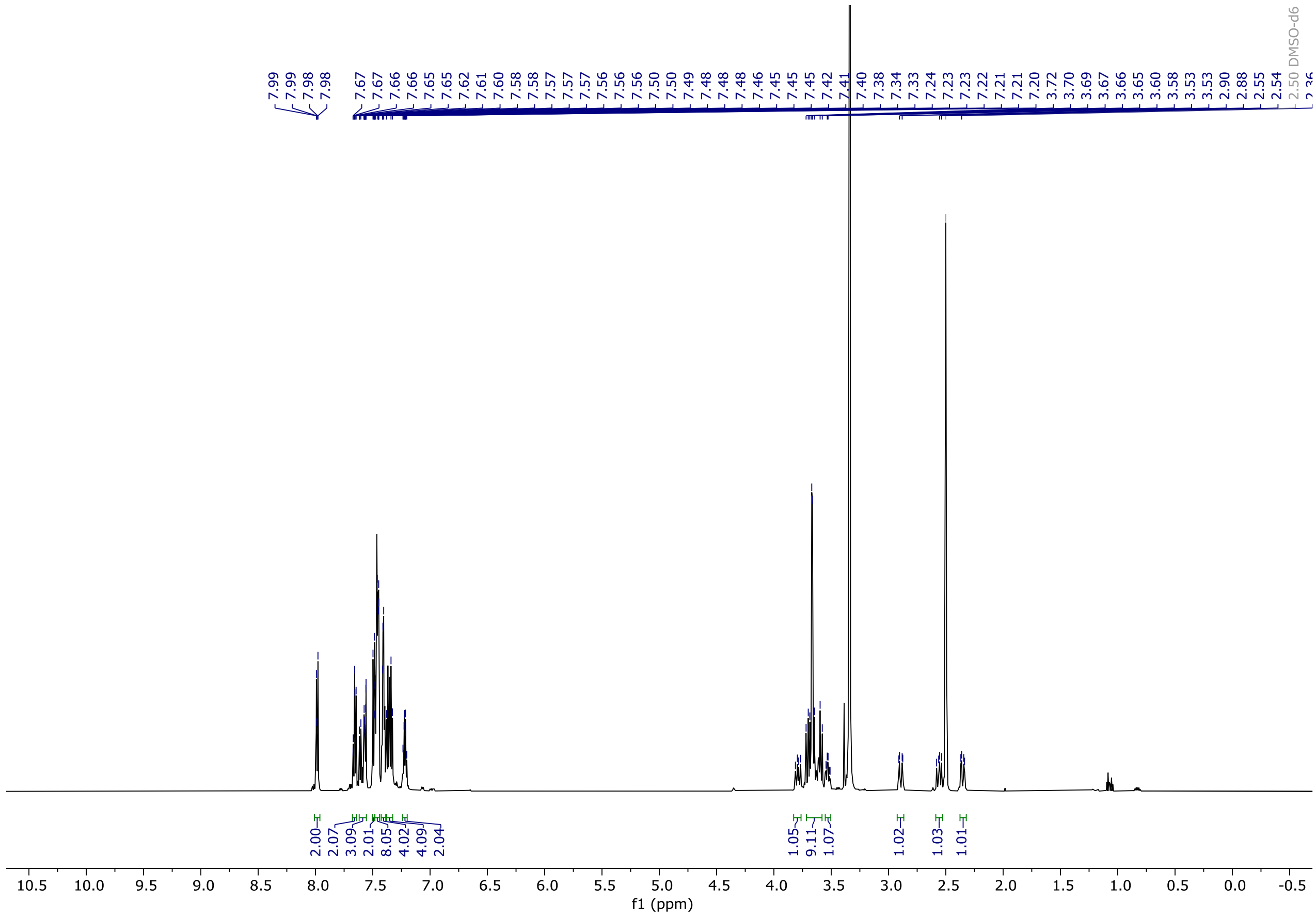

Figure S16. ^{1}H NMR spectrum of **9be** in DMSO-d_6

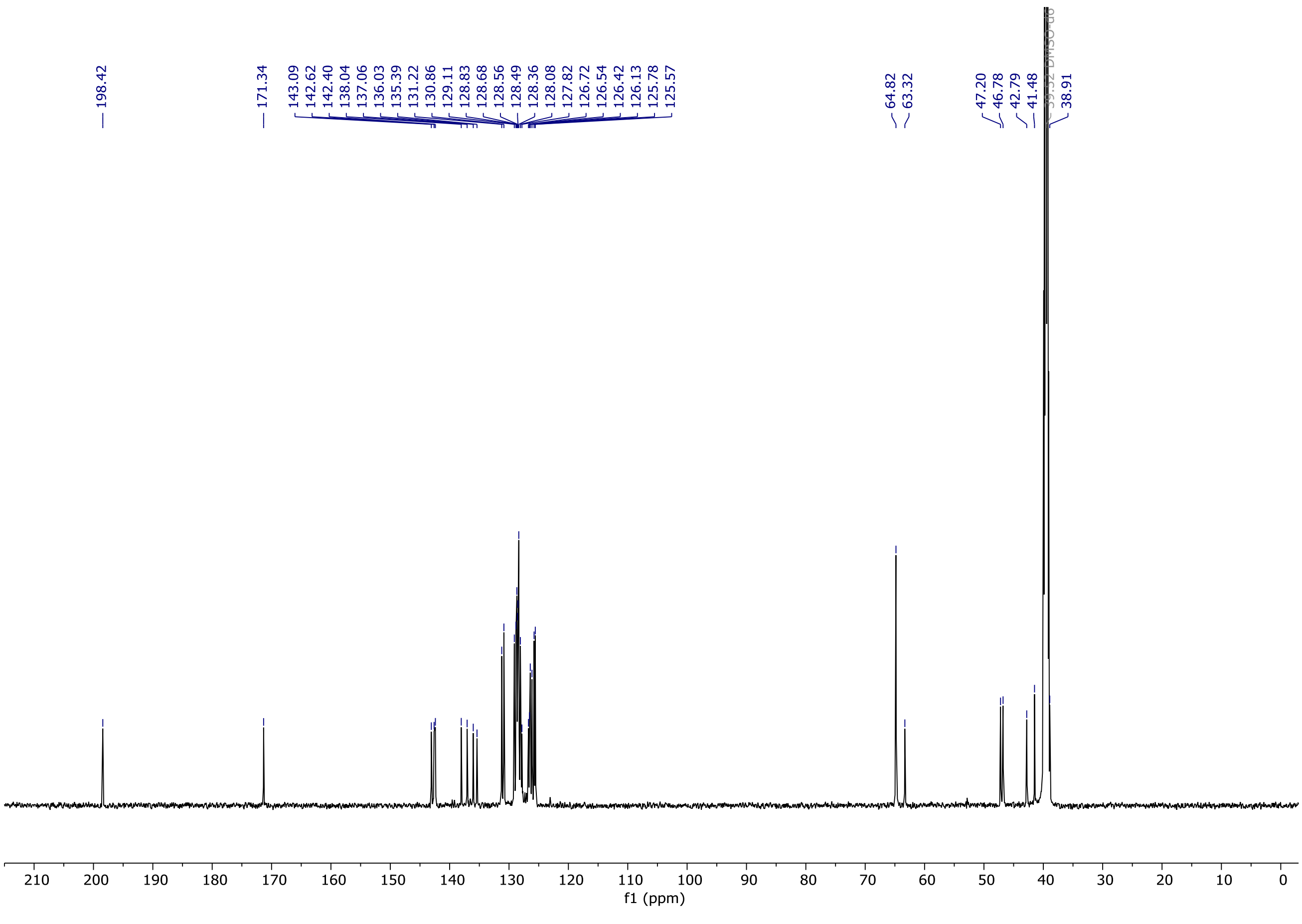

Figure S17. ^{13}C NMR spectrum of **9be** in DMSO-d_6

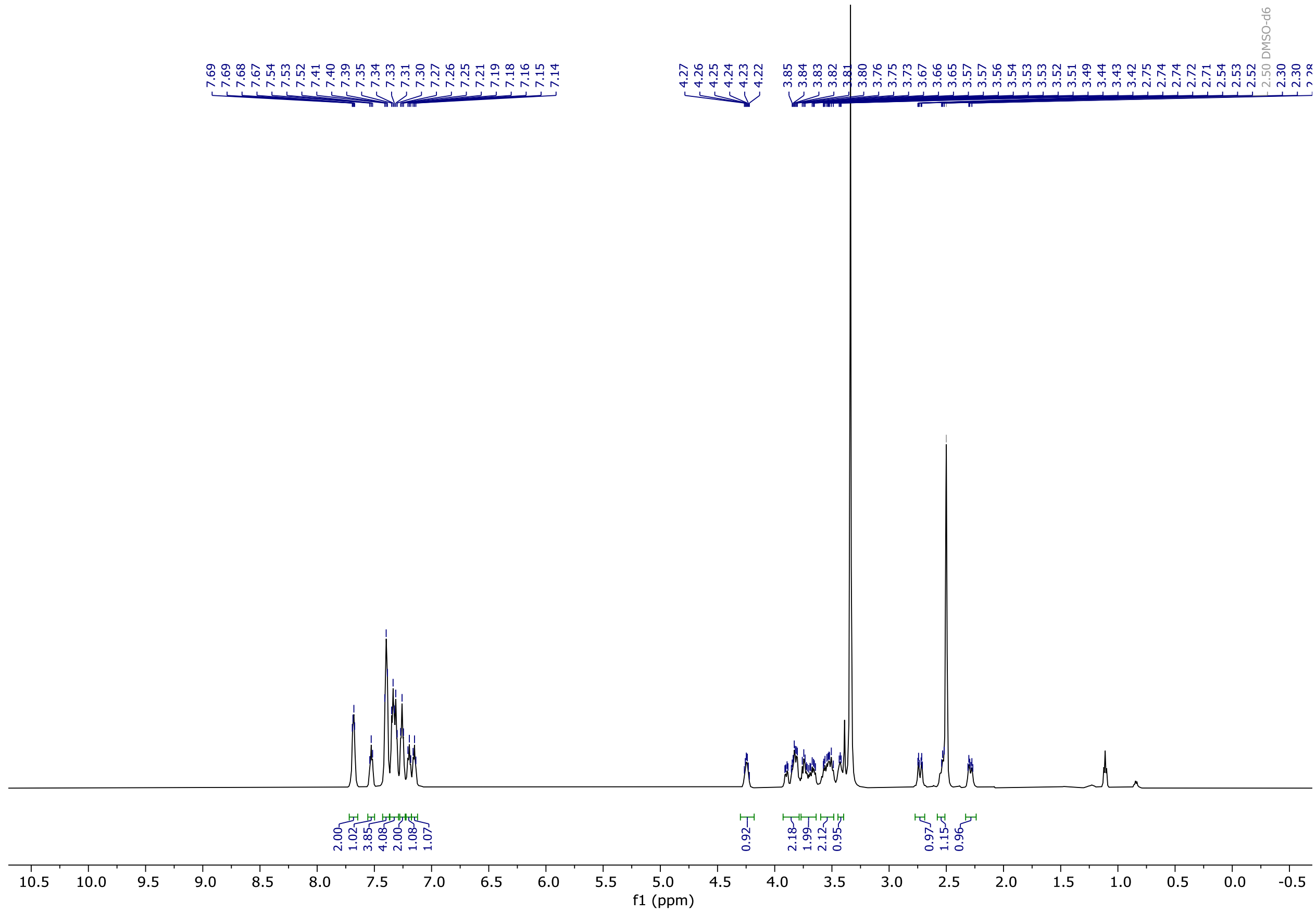

Figure S18. ^{1}H NMR spectrum of **9ac** in DMSO-d_6

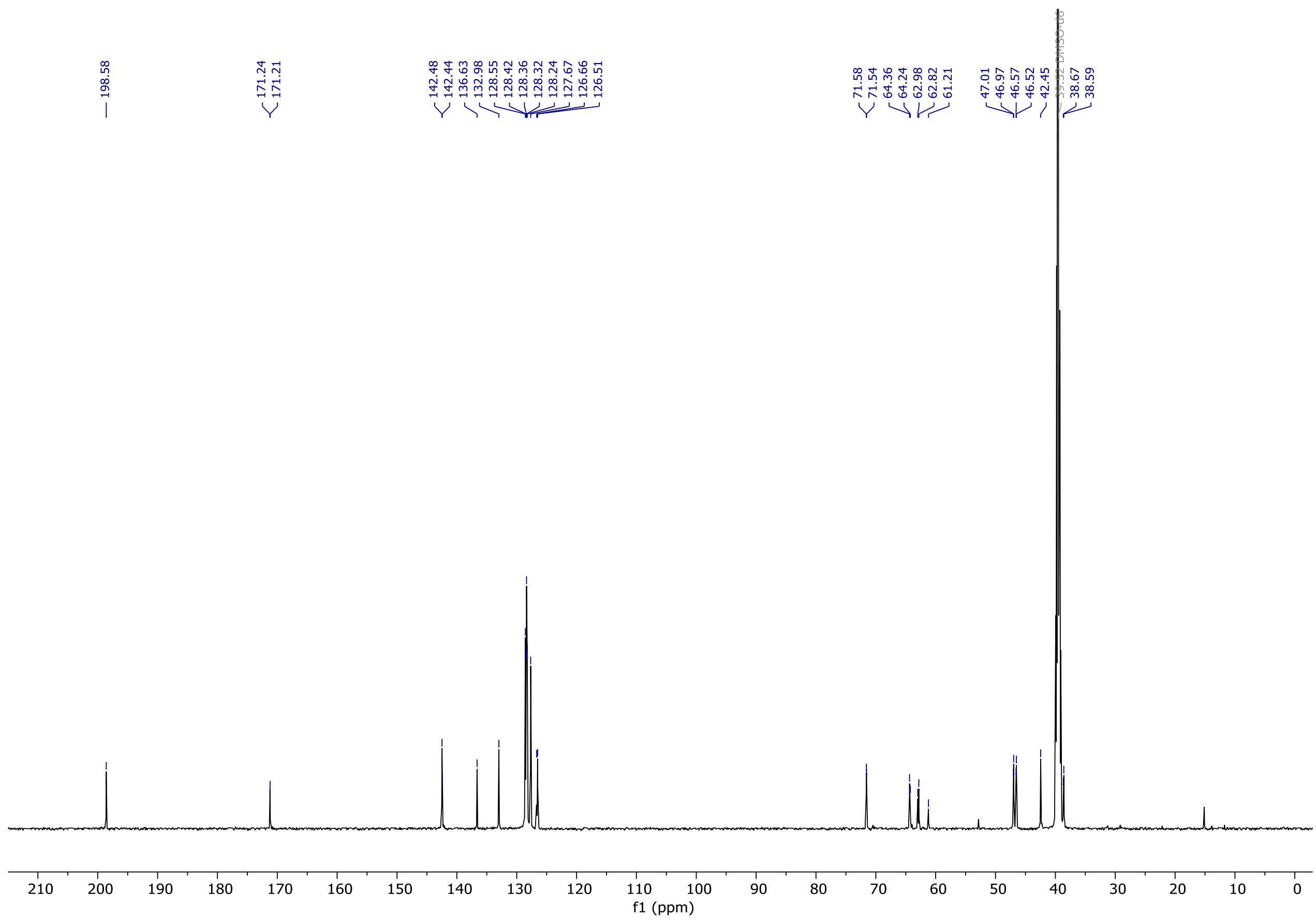

Figure S19. ^{13}C NMR spectrum of **9ac** in DMSO-d_6

6. FTIR analysis of **9bc** and its xerogels

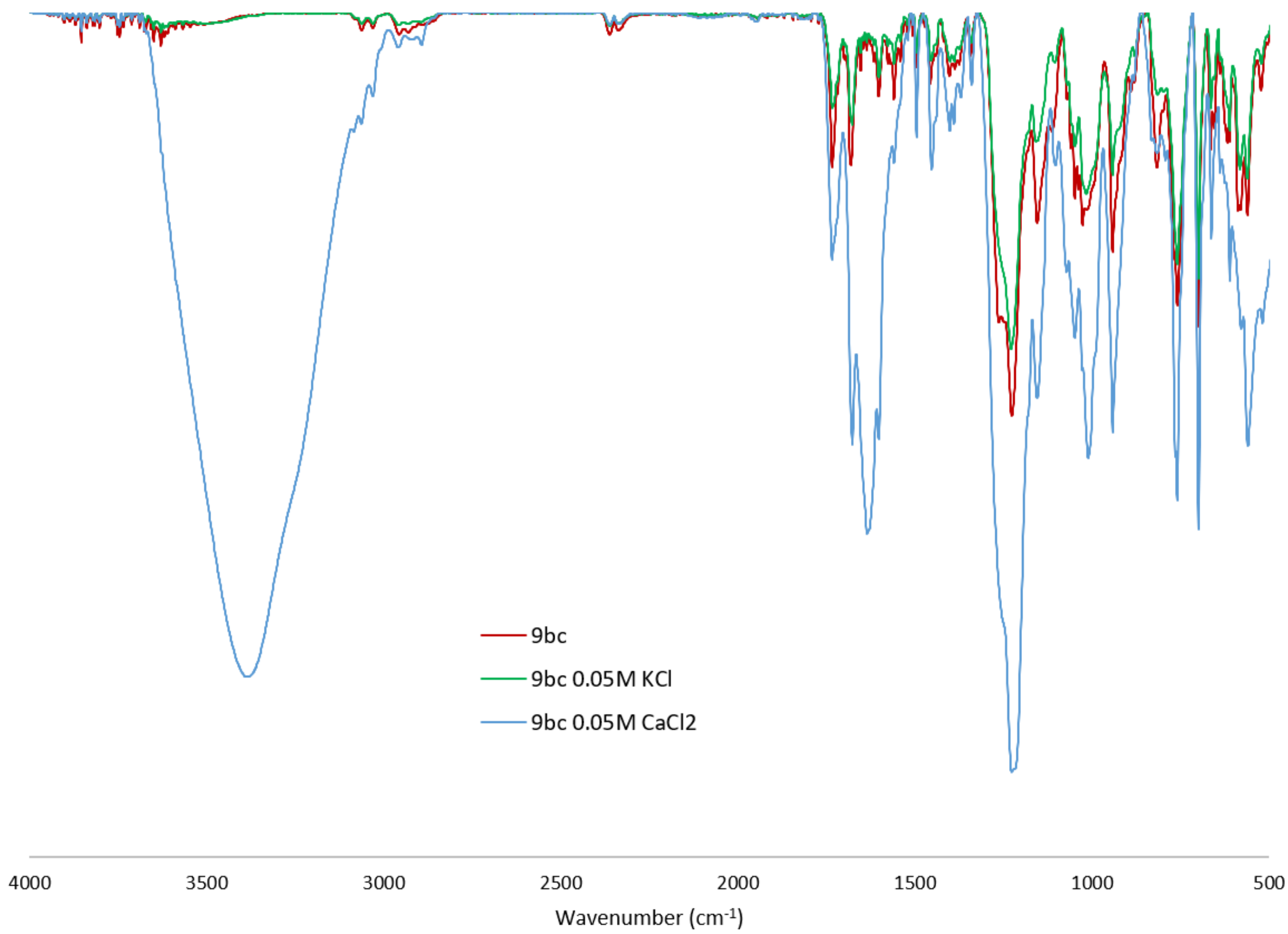

Figure S20. FTIR spectrum of **9bc** (red) and its 0.05M KCl (green) and $CaCl_2$ (blue) xerogels.
